# Extended insight into the *Mycobacterium chelonae-abscessus* complex through whole genome sequencing of *Mycobacterium salmoniphilum* outbreak and *Mycobacterium salmoniphilum*-like strains

**DOI:** 10.1038/s41598-019-40922-x

**Published:** 2019-03-14

**Authors:** Phani Rama Krishna Behra, Sarbashis Das, B. M. Fredrik Pettersson, Lisa Shirreff, Tanner DuCote, Karl-Gustav Jacobsson, Don G. Ennis, Leif A. Kirsebom

**Affiliations:** 1 Department of Cell and Molecular Biology, Box 596, Biomedical Centre, SE-751 24 Uppsala, Sweden; 2 Department of Biology, University of Louisiana, Lafayette, Louisiana, USA; 3 Department of Neuroscience, Box 593, Biomedical Centre, SE-751 24 Uppsala, Sweden

## Abstract

Members of the *Mycobacterium chelonae*-*abscessus* complex (MCAC) are close to the mycobacterial ancestor and includes both human, animal and fish pathogens. We present the genomes of 14 members of this complex: the complete genomes of *Mycobacterium salmoniphilum* and *Mycobacterium chelonae* type strains, seven *M. salmoniphilum* isolates, and five *M. salmoniphilum*-like strains including strains isolated during an outbreak in an animal facility at Uppsala University. Average nucleotide identity (ANI) analysis and core gene phylogeny revealed that the *M. salmoniphilum*-like strains are variants of the human pathogen *Mycobacterium franklinii* and phylogenetically close to *Mycobacterium abscessus*. Our data further suggested that *M. salmoniphilum* separates into three branches named group I, II and III with the *M. salmoniphilum* type strain belonging to group II. Among predicted virulence factors, the presence of phospholipase C (*plcC*), which is a major virulence factor that makes *M. abscessus* highly cytotoxic to mouse macrophages, and that *M. franklinii* originally was isolated from infected humans make it plausible that the outbreak in the animal facility was caused by a *M. salmoniphilum*-like strain. Interestingly, *M. salmoniphilum*-like was isolated from tap water suggesting that it can be present in the environment. Moreover, we predicted the presence of mutational hotspots in the *M. salmoniphilum* isolates and 26% of these hotspots overlap with genes categorized as having roles in virulence, disease and defense. We also provide data about key genes involved in transcription and translation such as sigma factor, ribosomal protein and tRNA genes.

## Introduction

Mycobacteria occupy various ecological niches and can be isolated from soil, tap water and ground water and they are divided into slow (SGM) and rapid (RGM) growing mycobacteria. Several cause diseases both in humans and animals (land and aquatic). Among these, *Mycobacterium tuberculosis* (*Mtb*) and *Mycobacterium leprae*, the causative agents of tuberculosis and leprosy, respectively, are well-known pathogenic mycobacteria. *Mycobacterium piscium* was the first *Mycobacterium* spp. to be isolated from fish^1^, however, it has been lost. Many different mycobacteria have since been isolated from various infected fish: the fish disease caused by mycobacteria is referred to as mycobacteriosis (fish tuberculosis). Infections are due to three predominant mycobacteria: *Mycobacterium marinum* (*Mma*); *Mycobacterium chelonae* (*Mche*); and *Mycobacterium fortuitum* (*Mfor*). Of these, the SGM *Mma* seems to be the most important species infecting a wide array of different fish, in particular in warm water systems, while the coldwater pathogen *Mche* infects predominantly salmonid species^2^. Several other mycobacteria such as *Mycobacterium abscessus* (*Mabs*) and *Mycobacterium salmoniphilum* (*Msal*) have emerged as fish pathogens. The RGM *Msal* belongs to the *Mche*-*Mabs* (MCAC) complex^3^ and, as *Mche* it causes mycobacteriosis in cold water living fish^4–6^. *Msal* was originally identified from salmonids^7^ but lost its species status in 1980 due to its high biochemical similarity with *Mche* and *Mabs*. On the basis of phylogenetic analysis of the 16S rRNA gene, *rpoB* and *hsp65* and mycolic acid composition *Msal* regained species status 2007^3,8^.

Mycobacterial infections are common among wild fish but it is most problematic in aquaculture and aquarium settings. To prevent and treat bacterial infections in aquaculture settings antimicrobial agents are used in large quantities worldwide as well as the use of medicated fish food^9^. In addition, the MCAC-complex contains many clinically relevant human pathogens but *Msal* has not been implicated to cause disease in humans^10,11^. Recently, the clinically isolated human pathogen *Mycobacterium franklinii* (*Mfra*) was classified as a mycobacterial species and member of the MCAC-complex^10,12^. As exemplified by the emerging pathogen *Mabs*, members of the MCAC-complex display resistance to many clinically relevant antibiotics and hence infections caused by these mycobacteria can be problematic and treatment requires the use of other antibiotics than in treatment of tuberculosis^11^. Together this imposes a potential risk for selecting antibiotic resistant microbes and thereby constitutes a threat to animal and human health^9,13–16^.

In 2012, there was an outbreak of a bacterial infection in the animal facility among the mice population at the Biomedical Center, Uppsala University. Subsequently, two different mycobacteria were isolated from the tap water in the animal facility on two different occasions. On the basis of partial 16S rRNA gene sequences and other biochemical tests (see acknowledgments) the single isolate from the first sampling was identified as *Msal*, while the three isolates from the second sampling were classified as *M*. *salmoniphilum*- (*Msal*-) like. However, it is still not clear whether the sampled mycobacteria caused the outbreak. Neither is it known if the isolates named as *Msal*-like are *Msal* strains or represent different species. On the basis of this and together with the importance of this group of mycobacteria with respect to pathogenicity, emerging antibiotic resistance and the phylogenetic closeness of MCAC-complex members to the mycobacterial ancestor^17,18^ (unpublished) provided the incentives for a comparative genomic analysis of these closely related mycobacterial species.

Here we present the complete genomes of the *Msal* DSM43276 (*Msal*^T^) and *Mche* DSM43804 (*Mche*^T^) type strains, seven *Msal* strains (including outbreak strains), and five *Msal*-like isolates. Our comparative genomic analysis, where we included the genomes of *Mabs*, and 36 additional MCAC-complex members, revealed that *Msal* and *Msal*-like strains represent two different species. Whole genome average nucleotide identity and core gene phylogeny further suggested that the *Msal*-like isolates should be referred to as *Mfra* strains and that they are phylogenetically close to *Mabs*. Our data further suggest that *Msal* constitute three separate groups.

## Results

### Overall description of the genomes

To understand the interrelationship between *Msal* and *Msal*-like strains we obtained strains from various sources including *Mche*^T^ (DSM43804; Table 1 and S1). The type strain *Msal*^T^ (DSM43276) formed both rough (R) and smooth (S) colony morphotypes (the other *Msal* strains formed R colonies). After re-streaking to obtain homogenous cultures the R type was used for genome sequencing (sequencing the 16S rDNA suggested that both types correspond to *Msal*; not shown). The collection also included *Msal*-like strains from the 2012 outbreak at the animal facility at the Uppsala University Biomedical Center and *Msal*-like strains isolated from tap water at different time points between 2011 and 2013 in Uppsala (Sweden). DNA from the different strains were isolated and subjected to sequencing (see Methods and Supplementary information).

**Table 1:** Summary of genome annotation.

Species	Name tag	Genome size(bp)	(%)GC content	Annotation (number of.)						Bioproject ID	Accession no	Source
				Scaffolds	CDSs	rRNAs (5S;23S;16S)	tRNAs	ncRNAs	signal peptides			
*M. salmoniphilum* CCUG60883	*Msal* CCUG60883	5076038	64	15	4950	(1;1;1)	51	38	458	PRJNA414709	PECM00000000	CCUG strain*
*M. salmoniphilum* CCUG60884	*Msal* CCUG60884	4963292	64,2	16	4832	(1;1;1)	56	36	429	PRJNA414709	PECL00000000	CCUG strain*
*M. salmoniphilum* CCUG60885	*Msal* CCUG60885	5076073	64	14	4953	(1;1;1)	51	38	455	PRJNA414709	PECK00000000	CCUG strain*
*M. salmoniphilum* CCUG62472	*Msal* CCUG62472	5176285	64,2	16	5061	(1;1;1)	57	34	435	PRJNA414709	PECJ00000000	CCUG strain*
*M. salmoniphilum* DE4585	*Msal* DE4585	5072347	64,1	11	4867	(1;1;1)	61	39	442	PRJNA414709	PECH00000000	Outbreak strain Ennis
*M. salmoniphilum* DE4586	*Msal* DE4586	4817070	64,5	31	4645	(1;1;1)	55	39	428	PRJNA414709	PECG00000000	Outbreak strain Ennis
*M. salmoniphilum* DE4587	*Msal* DE4587	4802997	64,2	11	4629	(1;1;1)	55	39	427	PRJNA414709	PECI00000000	Outbreak strain Ennis
***M. salmoniphilum*** **DSM43276**	***Msal*** ^**T**^	4776625	64,3	1	4652	(1;1;1)	56	31	414	PRJNA414709	CP024633	DSM strain**
*M. salmoniphilum-like* CCUG63695	*Msal-like* CCUG63695	4998469	64,2	14	4847	(1;1;1)	53	45	430	PRJNA414709	PECE00000000	CCUG strain*
*M. salmoniphilum-like* CCUG63696	*Msal-like* CCUG63696	4997587	64,2	9	4844	(1;1;1)	53	44	432	PRJNA414709	PECD00000000	CCUG strain*
*M. salmoniphilum-like* CCUG63697	*Msal-like* CCUG63697	5008405	64,2	36	4856	(1;1;1)	53	45	430	PRJNA414709	PECC00000000	CCUG strain*
***M. salmoniphilum-like*** **CCUG64054**	***Msal-like*** ^**CCUG64054**^	5011360	64,2	46	4866	(1;1;1)	53	45	432	PRJNA414709	PECB00000000	CCUG strain*
*M. salmoniphilum-like* CCUG64056	*Msal-like* CCUG64056	4998395	64,2	8	4844	(1;1;1)	53	45	430	PRJNA414709	PECF00000000	CCUG strain*
*M. franklinii* DSM45524	*Mfra* DSM45524^T^	5408993	64,1	34	5334	(1;1;1)	53	45	474	PRJNA509866	RXLR00000000	DSM strain**
***M. chelonae subsp. chelonae*** **DSM43804**	***Mche*** ^**T**^	5030282	63,9	1	4894	(1;1;1)	47	34	445	PRJNA508902	CP034383	DSM strain**
***M. abscessus*** **ATCC19977**	***Mabs*** ^**ATCC19977**^	5067172	64,1	1	4955	(1;1;1)	47	51	440	PRJNA61613	NC_10397	NCBI:NC_10397

Summary of genome annotation and sources of *Msal* and *Msal-*like strains, *Mche*^T^ and *Mabs*^ATCC19997^.

Note: All genomes were annotated using PROKKA pipeline. CDS: Coding Sequences (Prodigal), rRNA: ribosomal RNA (rnammer), tRNA: Transfer RNA (tRNAScanSE), ncRNA: non-coding RNA (Rfam) and signal peptides (signalp); Species highlighted in bold are representative genomes in this article.

*Strains obtained from the CCUG strain collection, Goteborg, Sweden; **Strains obtained from the Deutsche Sammlung von Mikroorganism and Zellkulturen, Germany.

**Figure 1:**
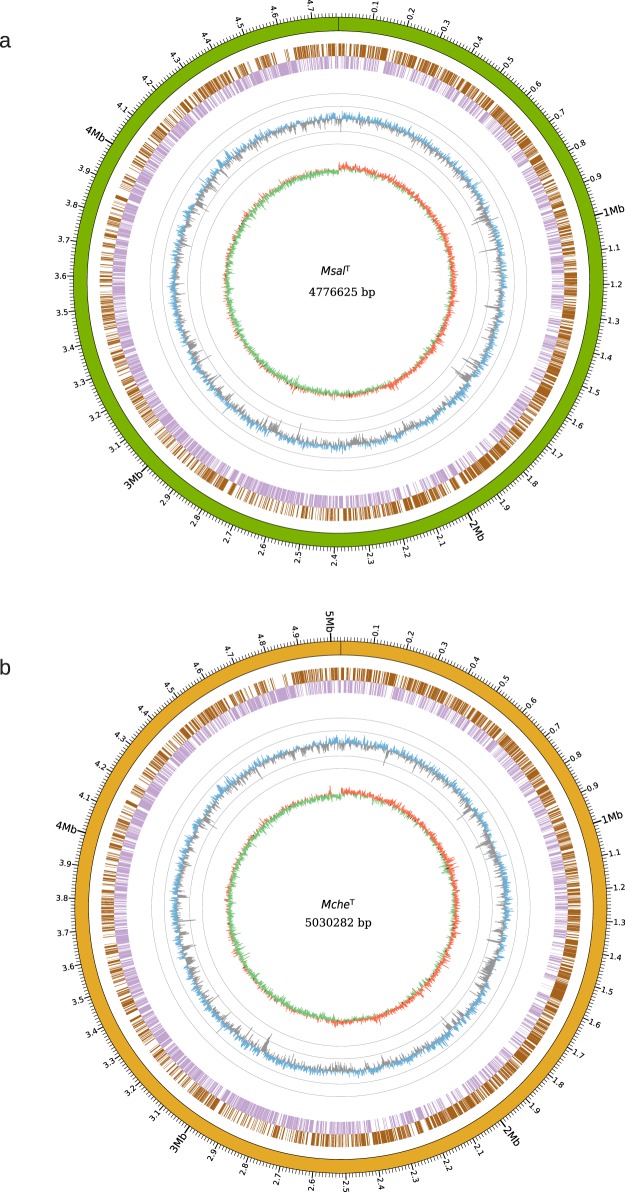
Overview of the *Msal*^T^ and *Mche*^T^ genomes. (**a**) Circos plot showing the complete genome sequence of *Msal*^T^. From outer to inner circle: Green track represents the complete genome overlapping with scale along the genome length. The next two circles, marked as brown and violet blocks, represent genes in forward (brown) and reverse (violet) strands. The circle with blue (higher than the mean value) and grey (lower than the mean value) “spikes” show the GC-content distribution calculated using a sliding window of 1000 bp, while each grey circle represent variations of the mean GC-content 64.3% in ±10 and ±20 units (*i.e*. outer grey circle = 84.3% and inner grey circle = 44.3%). The inner track in red (positive) and green (negative) circle shows the GC-skew using a sliding window of 1000 bp. (**b**) Same as in (**a**) for *Mche*^T^ where the complete genome overlapping with scale along the genome length (outer circle), which is illustrated in dark yellow. The mean GC-value equals to 63.9%.

*De novo* assembly of the long Pac-bio reads (average length 10 kbp) with a coverage of 100x resulted in single scaffolds (one contig for each genome) representing the complete *Msal*^T^ and *Mche*^T^ genomes (4,776,625 and 5,030,282 bps, respectively; Fig. 1 and Table 1). The average GC-contents were calculated to be 64.3% and 63.9%, respectively. We predicted that the *Msal*^T^ and *Mche*^T^ genomes encompass 4712 and 4945 genes. Of these, 4652 (4894 for *Mche*^T^) correspond to coding sequences (CDS), 56 tRNA genes (*Mche*^T^ 47), one rRNA operon (gene order; 16S, 23S, and 5S rRNA) and one transfer-messenger RNA (tmRNA; Table 1). The genome-wide distribution of the tRNA genes is shown in Fig. S1. Of note, *Msal*^T^ and *Mche*^T^ encode two tRNA^Cys^ and two (*Mche*^T^ one) tRNA^His^ isoacceptors as previously observed in other mycobacteria^19,20^ (see below; Behra *et al*. unpublished).

For the other strains, seven *Msal* and five *Msal*-like strains (Table 1; Fig. S2), the average genome coverage of the Illumina reads ranged from 250x to 600x. The reads were assembled into high quality, near complete genomes (approx. 95% complete) supported by high N50 values and few scaffolds (Table 1; Fig. S2). The sizes of the assembled draft genomes vary from 4,802,997 to 5,176,285 base pairs (Table 1). The average GC-content for these strains was determined to be similar as the GC-content calculated for *Msal*^T^ and *Mche*^T^ (Table 1). Predicted number of CDS varies between 4629 and 5061 consistent with their genome sizes, while the number of tRNA genes range between 47 and 61 (see below). As for *Msal*^T^ and *Mche*^T^, one rRNA operon and one tmRNA gene were detected (Table 1 and S2). The number of predicted non-coding RNAs (ncRNAs) in *Msal* varies from 31 to 39, while all *Msal*-like (except *Msal*-like^CCUG63696^) strains were predicted to encode for 45 ncRNAs (Table 1 and S2). The higher number in the *Msal*-like strains is due to the presence of extra copies of genes encoding Ms_IGR8 (Table S2). For *Mche*^T^ and *Mabs*^ATCC19977^ we predicted 34 and 51 ncRNA genes, respectively (Table 1 and S2).

Whole genome alignment of the complete genomes *Msal*^T^, *Mche*^T^, *Mabs*^ATCC19977^ and the draft genome *Msal*-like^CCUG64054^ revealed high homology. Short inversions were detected in *Mabs*^ATCC19977^ and *Msal*-like^CCUG64054^ compared to *Msal*^T^ and *Mche*^T^ (Fig. S1a).

Except for *Msal*^T^ (Table 1), we could not identify any plasmid sequences in either of the genomes including *Mche*^T^. Presence of a low number (one or two) of incomplete and intact phages, on the other hand, was predicted in all the genomes. The phage sequences constitute less than one percent of the genome size irrespective of strain, except for *Msal*^CCUG62472^ where the phage sequences covers approx. 2.2% (Fig. S3; Table S3). A comparison with other MCAC-members (*Msal*, and *Mfra* strains; see below) revealed that the fraction of phage sequences for the *Mfra*^DSM45524^ isolates was higher (≈3%; Fig. S3; Table S3; of note, these MCAC strains were also predicted to carry phages classified as questionable). For *Mche*^T^ and *Mabs*^ATCC19977^ approx. 1.5% of their genomes represent predicted phage sequences.

For *Msal*^T^ two insertion sequence (IS) elements belonging to the ISAs1 and IS701 families were detected. These two IS elements were also detected in the *Msal*^DE4585–4587^ isolates, while the *Msal*^CCUG^-isolates were predicted to have additional IS elements belonging to other families (Table S4). For the *Msal*-like^CCUG^ strains we also detected ISAs1 and IS701 and the presence of an additional IS element, ISL3 (two copies in *Msal*-like^CCUG63697^). The ISAs1 and IS701 elements are present in other MCAC-members including *Mche*^T^ and *Mabs*^ATCC19977^ (see below). For these isolates, we also detected other IS element families, in particular different *Mfra* and *M. sp*. strains (which cluster together with *Msal*, see below) carry significantly higher numbers compared to our *Msal* or *Msal*-like strains. The total number of IS elements varied between two and 54 with *Mche*^T^ having three, while *M. sp*. D16Q20 carries 54 belonging to 14 different types and *Mabs*^ATCC19977^ a total of six (five types) IS elements (Table S4).

### Average nucleotide identity (ANI) analysis reveals that *Msal* and *Msal*-like strains cluster into two groups

**Figure 2:**
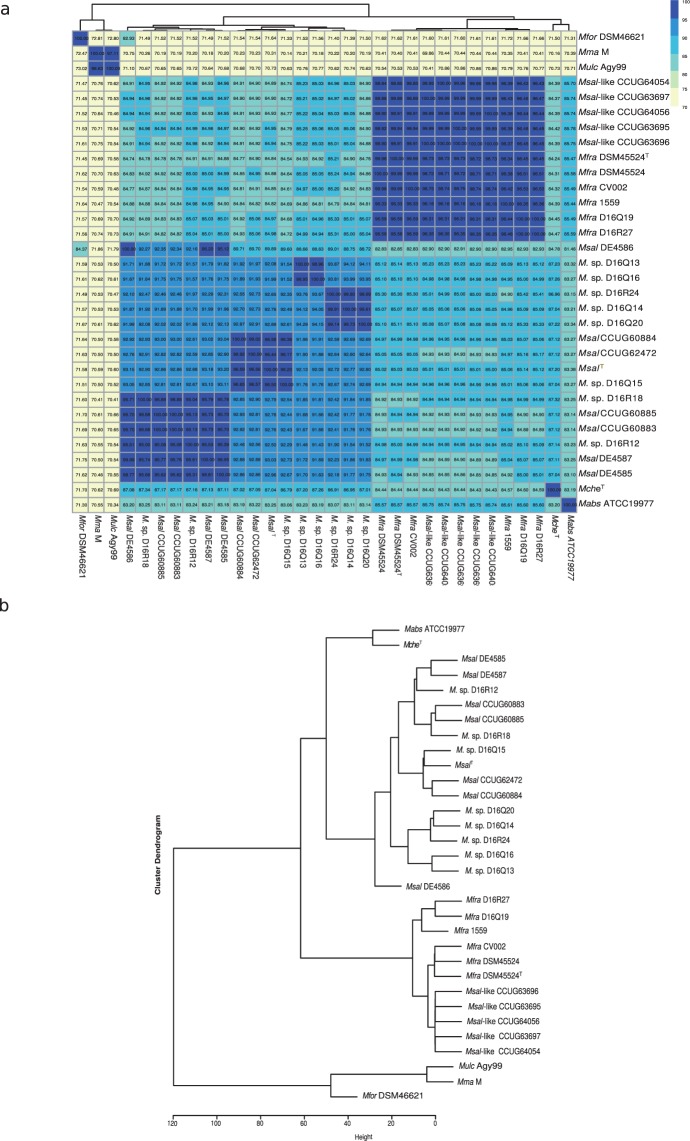
Clustering of *Msal*, *Msal*-like and other MCAC-members based on the average nucleotide scores (ANI) as indicated. (**a**) Heat map showing ANI values for ‘all-versus-all’ *Msal* and *Msal*-like strains including other members of the MCAC. ANI values were clustered based on unsupervised hierarchical clustering (see Methods). The horizontal tree represents the heatmap clustering of column wise dendogram. (**b**) Dendogram, extracted from the heat map in (**a**), showing clustering of different strains/isolates based on ANI values.

Unsupervised hierarchical clustering of the “all-versus-all” ANI scores clustered *Msal* and *Msal*-like into different groups. The ANI values for the *Msal* and *Msal*-like strains varied between 84–87% (Fig. 2a,b), significantly lower than the threshold 95%^20,21^ to be considered to belong to the same species. Moreover, the *Msal* strains can be sub-divided into groups (see also below); group I, *Msal*^CCUG60883^, *Msal*^CCUG60885^, *Msal*^DE4587^, and *Msal*^DE4585^, group II, *Msal*^T^, *Msal*^CCUG62472^, and *Msal*^CCUG60884^. The ANI scores between “intra-group” members are >95%, while for “inter-group” members it is ≈92% (Fig. 2a,b). The outbreak strain *Msal*^DE4586^ could not be referred to any of these two groups (but see below). Moreover, *Msal* strains are closer (ANI ≈ 87%) to *Mche*^T^, while the *Msal*-like strains cluster close to *Mabs* (ANI >85%; Fig. 2a).

Expanding the ANI analysis by including 14 MCAC-members, for which the genomes are publicly available (Table S1; ftp://ftp.ncbi.nlm.nih.gov/genomes/; last accessed Aug 2017; see also ref.^22^), revealed that the *Msal*-like strains cluster together with *Mfra*; ANI scores higher than 96% (Fig. 2a,b; of note, *Mfra*^CV002^, *Mfra*^DSM45524^ and *Mfra*^DSM45524T^ are the same *Mfra* strain but represent draft genomes sequenced in different laboratories where the *Mfra*^DSM45524T^ draft genome was sequenced in connection with the present study, see Table S1). These data also suggested that the *Mycobacterium* sp. D16 strains cluster together with *Msal* group I (D16R12 and D16R18), *Msal* group II (D16Q15) or close to these two groups (D16Q13, D16Q14, D16Q16, D16Q20 and D16R24). Hence, this extended analysis suggested that *Msal*^D16Q13^, *Msal*^D16Q14^, *Msal*^D16Q16^, *Msal*^D16Q20^ and *Msal*^D16R24^ constitute a third *Msal* group, group III.

To conclude, these data suggested that *Msal* and *Msal*-like strains cluster in distinct groups. Moreover, the *Msal* strains cluster into three groups separating them from *Mche*^T^ as expected.

### Core genes and comparative analysis

**Figure 3:**
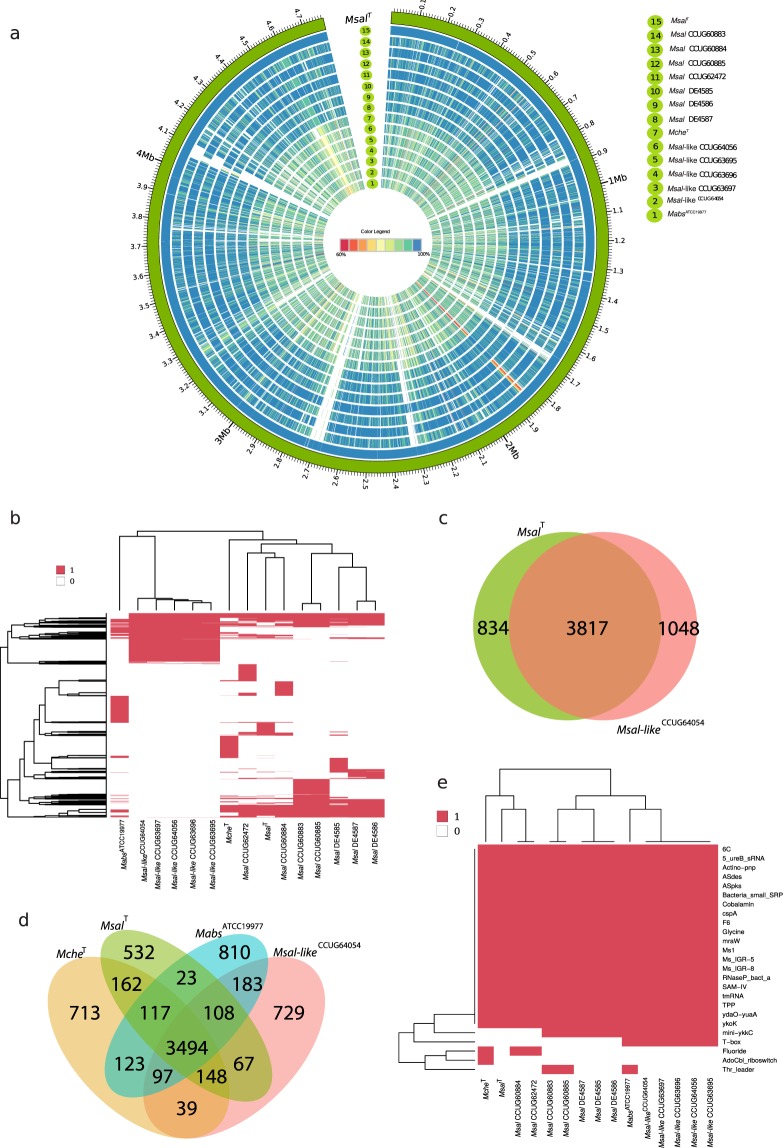
Comparative analysis of orthologous genes predicted to be present in *Msal* and *Msal*-like strains, *Mche*^T^ and *Mabs*^ATCC19977^. (**a**) Circos plot showing the presence of protein coding genes in seven *Msal*, five *Msal*-like strains, *Mche*^T^ and *Mabs*^ATCC19977^ compared to the reference genome *Msal*^T^. The outer track (green) represents the genome for *Msal*^T^ with a size scale, while the next circle in blue corresponds to the predicted protein coding sequences (CDS) for *Msal*^T^. Subsequent circular tracks represent one genome and the number corresponds to the strain name in the legend on the right. Colored radial blocks represent orthologous genes in the corresponding genome and color intensity (see color scale in the middle) indicates percentage identity at the protein level. The white blocks indicate that no orthologs were identified. (**b**) Heat map showing presence (red) and absence (white) of orthologous genes (excluding core genes) mapped in different *Msal* and *Msal*-like strains, *Mche*^T^ and *Mabs*^ATCC19977^, and clustered using hierarchical clustering. The horizontal and vertical trees represent the heat map clustering of the column and row wise dendograms. (**c**) Venn diagram showing common and unique coding genes for *Msal* and *Msal*-like representative strains as indicated. (**d**) Venn diagram showing common and unique genes in *Msal*^T^, *Msal*-like^CCUG64054^, *Mche*^T^ and *Mabs*^ATCC19977^. (**e**) Heat map showing presence (red) and absence (white) of orthologous ncRNA genes mapped as in (**b**). The ncRNA genes were identified using Rfam, see main text. The horizontal and vertical trees represent the heat map clustering of the column and row wise dendograms.

To further understand the interrelationship between *Msal* and *Msal*-like strains (in total 12 strains) and their relation to *Mche*^T^ and *Mabs*^ATCC19977^ we used the complete *Msal*^T^ genome sequence as a reference genome for identification of orthologous protein coding sequences (CDS). Using amino-acid percentage identity for these CDS clustered *Msal* and *Msal*-like strains into two groups in keeping with the ANI data (Fig. 3a; see above). For the *Msal* strains (relative *Msal*^T^) the amino-acid percentage identity for the majority of CDS was ≥95% (Fig. S4a), while for the *Msal*-like strains it ranged between 85% and 95%. Comparing amino-acid percentage identity for *Msal*^T^, *Mche*^T^ and *Mabs*^ATCC19977^ revealed that *Msal*^T^ and *Mche*^T^ show higher similarity than *Msal*^T^ and *Mabs*^ATCC19977^ (Fig. S4a).

To further investigate the variation in gene content across the *Msal* and *Msal*-like strains, we identified core and unique genes where core genes are the set of genes present in all genomes. This analysis also revealed sets of genes predicted to be present in either *Msal* or *Msal*-like strains (Fig. 3b). In total 3817 CDS were present in both *Msal*^T^ and *Msal*-like^CCUG64054^, while 834 and 1048 unique CDS were identified in *Msal*^T^ and *Msal*-like^CCUG64054^, respectively (Fig. 3c). One may argue that this analysis was biased since we compared the *Msal*^T^ complete genome with the draft *Msal*-like^CCUG64054^ genome. But, since the *Msal*-like^CCUG64054^ genome is estimated to be 95% complete the numbers of unique genes that are false positive are probably few. Including *Mabs*^ATCC19977^ and *Mche*^T^ in this analysis predicted that 3494 core CDS are present in *Msal*^T^, *Msal*-like^CCUG64054^, *Mabs*^ATCC19977^ and *Mche*^T^ (Fig. 3d). For functional classification of CDS (including core and unique genes) in selected species see below.

Considering ncRNAs, while the majority of the Rfam annotated ncRNA genes were predicted to be present in these MCAC-members, the T-box category appears to be missing in *Msal* strains and *Mche*^T^ (Fig. 3e; T-boxes are riboswitches present in the leader region of genes/operons in bacteria influencing their expression^23^). Sequence alignment of the *ileS* gene revealed a putative T-box upstream of *ileS* in all MCAC-members (Fig. S4b). However, we did detect structural variations comparing *ileS* T-boxes originating from MCAC-members and *Mycobacterium smegmatis* (*Msmeg*) MC^2^155, in particular with respect to the S-turn in stem II (Fig. S4c)^24,25^.

### Phylogenetic analysis

**Figure 4:**
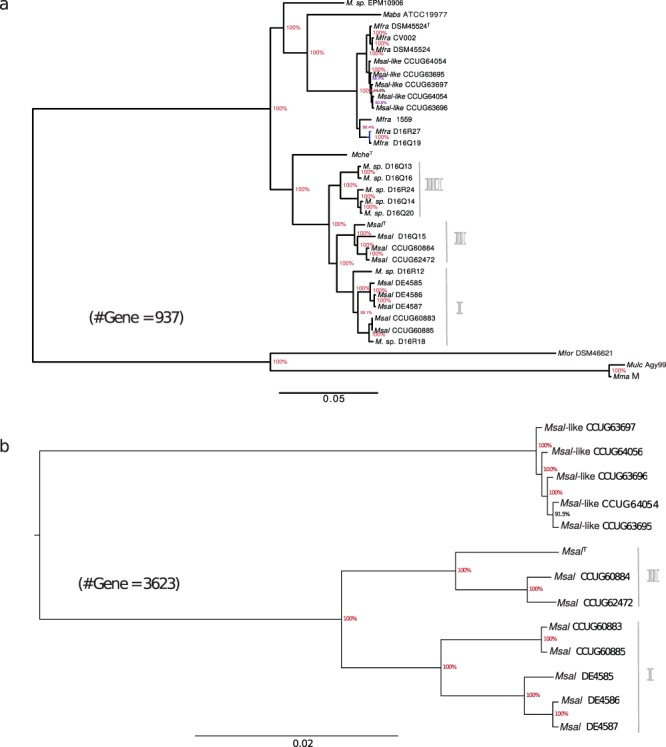
Phylogenetic relationship of *Msal* and *Msal*-like strains, *Mche*^T^ and *Mabs*^ATCC19977^. (**a**) Phylogenetic tree based on 937 core genes present in *Msal*, *Mfra* (*Msal*-like) strains, *Mche*^T^, *Mabs*^ATCC19977^, *Mfor*^DSM46621^, *Mulc*^Agy99^ and *Mma* (M strain). For details see Methods. (**b**) Phylogenetic tree based on 3623 core genes present in *Msal* and *Msal*-like strains as indicated.

The data presented above suggest that *Msal* and *Msal*-like strains cluster into two groups. Hence, we generated a core gene phylogenetic tree where we used 937 core genes (see above) present in the 13 *Msal* and *Msal*-like strains, *Mche*^T^, *Mabs*^ATCC19977^, and the 14 MCAC-members for which genomes are available (see above and Table S1). As outgroups, we used *Mfor*^DSM46621^, *Mma* (M strain^26^) and *Mycobacterium ulcerans* Agy99 (*Mulc*^Agy99^). The resulting tree clustered the *Msal* and *Msal*-like strains in separate branches and suggested that the *Msal* strains share a common ancestor with *Mche*^T^, whereas the *Msal*-like strains are more closely related to *Mabs*^ATCC19977^ and clustered together with *Mfra* strains [Fig. 4a; of note, a tree based on complete 16S rRNA gene sequences displayed two main branches and it did not discriminate the *Msal*-like strains and *Mche*^T^ (Fig. S5a)]. This clustering into two separate branches is in keeping with the ANI data (see above) as is the tree based on 3623 core genes present in 12 *Msal* and *Msal*-like strains, and *Msal*^T^ (Fig. 4b). Moreover, consistent with the ANI data the *Msal* strains cluster together with *Mycobacterium* sp. D16 isolates into at least three groups close to *Mche*^T^ (Fig. 4a; for further details with respect to *M. sp*. D isolates, see ref.^22^).

Another member of MCAC is *Mycobacterium immunogenum* (*Mimm*) and recently the genome sequences for several *Mimm* and *Mycobacterium* spp. isolates were published (Table S1; ftp://ftp.ncbi.nlm.nih.gov/genomes/; last accessed Aug 2017). We therefore expanded our analysis by including these genomes and generated a core gene phylogenetic tree based on 623 genes present in these strains, *Mche*^T^, *Mabs* subsp. *bolletii*, *Mabs* subsp. *massiliense*, *Msal* and *Msal*-like strains (using the same outgroups as above). This tree displayed high bootstrap values and revealed that *Mimm* and *Mabs* shared a common ancestor and again clusters *Msal* and *Msal*-like strains in separate branches. In addition, this analysis suggested that *Mche*^T^ and several of the *Mycobacterium* spp. (*M. sp*. strains) H-strains shared a common ancestor and are grouped into three branches. The other H strains cluster together with *Mimm* (Fig. S5b). We conclude that *Msal*-like strains diverged before the separation of *Mimm* and *Mabs*.

### Functional classification of core and unique genes in *Msal*^T^, *Msal*-like^CCUG64054^, *Mche*^T^ and *Mabs*^ATCC19977^

**Figure 5:**
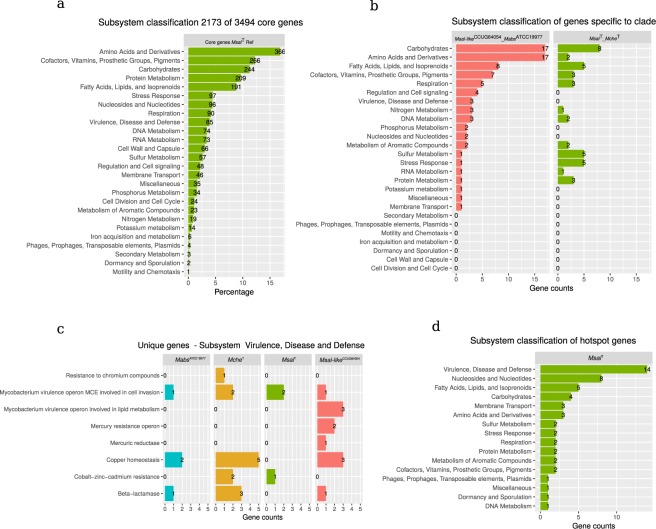
Functional classification of genes in *Msal*^T^, *Msal*-like^CCUG64054^, *Mche*^T^ and *Mabs*^ATCC19977^ into subsystem as indicated. (**a**) Subsystem classification of 2173 core genes using *Msal*^T^. Of note, that a gene can be classified in more than one subsystem. (**b**) Subsystem classification of specific genes present in *Mfra*^CCUG64054^ and *Mabs*^ATCC19977^, and present in *Msal*^T^ and *Mche*^T^ as indictated. (**c**) Classification of unique genes present in *Mabs*^ATCC19977^, *Mche*^T^, *Msal*^T^ and *Msal*-like^CCUG64054^ in the subsystem “Virulence, Disease and Defence”. (**d**) Subsystem classification of mutational hotspot genes in *Msal*, see main text for details.

We used the RAST subsystem and classified the function of 3494 core genes, present in *Msal*^T^, *Msal*-like^CCUG64054^, *Mche*^T^ and *Mabs*^ATCC19977^, and unique genes, which range between 532 and 810 genes (Fig. 3d; Table S5). This analysis revealed that 62.2% (2173) of the core genes could be classified into different subsystems (Fig. 5a). Considering the total number of CDS in these mycobacteria, the fraction in percentage of functionally classified genes was lower (ranging between approx. 52% and 57%), while the fraction of hypothetical genes was ≈30%. For genes classified into subsystems, the distribution of genes belonging to different categories was similar comparing *Msal*^T^, *Msal*-like^CCUG64054^, *Mche*^T^ and *Mabs*^ATCC19977^ (Fig. S6a).

Comparing functional classification of CDS unique (Fig. 3d) to the *Msal*^T^/*Mche*^T^ (162 genes) and *Msal*-like^CCUG64054^/*Mabs*^ATCC19977^ (183 genes) pairs showed that the fraction unique genes belonging to in particular the subsystems “Amino acids and Derivatives” and “Carbohydrates” was higher in *Msal*-like^CCUG64054^/*Mabs*^ATCC19977^, while *Msal*^T^/*Mche*^T^ carry higher numbers of genes involved in “Sulfur Metabolism” and “Stress Response” (Fig. 5b). Of note, *Msal*-like^CCUG64054^ and *Mabs*^ATCC19977^ also have four unique genes involved in “Regulation and Cell signaling”; classified as transcriptional regulator *whiD*, *hca* operon transcriptional activator, HTH-type transcriptional regulator *cynR*, and carbonic anhydrase 1 gene. However, *Msal*^T^/*Mche*^T^ carry other genes annotated as the three former genes whereas no carbonic anhydrase 1 gene could be detected in *Msal*^T^/*Mche*^T^ (see Discussion). Moreover, *Msal*-like^CCUG64054^/*Mabs*^ATCC19977^ carry three unique copper homeostasis genes (*copB*, *copZ* and *copA* homologs) belonging to the “Virulence, Disease and Defence” subsystem, while *Msal*^T^/*Mche*^T^ appears to have no unique genes in these two latter categories. But analyzing the four species separately, unique genes involved in copper homeostasis were predicted to be present in all species except for *Msal*^T^ (Fig. 5c).

Furthermore, functional classification of unique genes revealed that all four species carry genes in almost all subsystems (Fig. S6b; Table S5b–e). For example, *Mabs*^ATCC19977^ has a high number of unique genes in the “Amino acid and Derivatives” subsystem, while *Msal*-like^CCUG64054^ has higher numbers in the “Carbohydrate” subsystem. In these two subsystems, we noted the presence of unique genes in these four mycobacteria that are involved in the metabolism of specific amino acids and carbohydrates (Fig. S6c,d). In the “Fatty Acids, Lipids, and Isoprenoids” subsystem we observed differences in the presence/absence and copy number variation of genes comparing *Mabs*^ATCC19977^, *Mche*^T^, *Msal*^T^, and *Msal*-like^CCUG64054^ genomes (Fig. S6e). For example, the long-chain fatty acid CoA ligase *fad*13 gene, required for maintaining the appropriate mycolic acid composition and permeability of the cell wall^27,28^, was predicted to be present in nine copies in *Msal*-like^CCUG64054^. Of these nine copies, five were also predicted to be present in *Msal*^T^. Interestingly, the number of unique genes belonging to “Virulence, Disease and Defence” appears to be higher in *Mche*^T^ and *Msal*-like^CCUG64054^ relative to *Mabs*^ATCC19977^ and *Msal*^T^ (Fig. S6b).

### Identification of SNVs and mutational hotspots in *M. salmoniphilum* strains

Single nucleotide variations (SNVs) for the *Msal* genomes were predicted using *Msal*^T^ as reference and the program MUMmer^29^. The number of SNVs ranged between 136702 and 291755 for the different *Msal* strains. Mutational hotspots, which are genomic regions where the SNV frequencies are higher relative to the background, were identified for *Msal*. Application of the method described by Das *et al*.^30^ revealed 69 mutational hotspot regions in *Msal* with a high average number of SNVs (>150) per region (Fig. S6f). This corresponds to a frequency of 14.7/Mb. 168 genes overlap with the 69 hotspot regions and of these, 49 were annotated as hypothetical genes and 53 genes were classified into different subsystem categories (Fig. 5d; Table S6). Of the classified genes, >25% were predicted to belong to the category “Virulence, Disease and Defence” with several categorized as *mce* related (mammalian cell entry; Table S6). Interestingly, the ESX-1 associated gene *espR* is among the genes that overlap with the hotspot regions (see Discussion).

### Horizontal gene transfer, HGT

The total number of putative horizontally transferred genes ranged from 251 (*Msal*^DE4587^) to 345 (*Mfra*^DSM45524T^; Fig. S7a; Table S7). Of these, 66 were predicted to be present in *Mabs*^ATCC19977^, *Mche*^T^, *Msal*^T^ and *Msal*-like^CCUG64054^ (Fig. S7b; Table S7). Among possible donors of the HGT genes, members of the order *Streptomycetales*, *Micrococcales, Propionibacteriales, Streptosporangiales* and *Pseudonocardiales* were predicted to be the most likely donors (Fig. S7c). The gene annotations and function of the HGT genes are presented in Table S7. Among the HGT genes one was predicted, the lactate 2-monooxygenase gene, to be of eukaryotic origin and derived from the fungi *Ascomycota*. Mann-Whitney-Wilcoxon test with respect to GC-content (version R v3.2.2^31^) suggested that the GC-content deviates from the average GC-contents in the majority of the cases (Table S7) supporting the notion that candidate HGT genes have been horizontally transferred.

### Virulence genes and ESX genes

**Figure 6:**
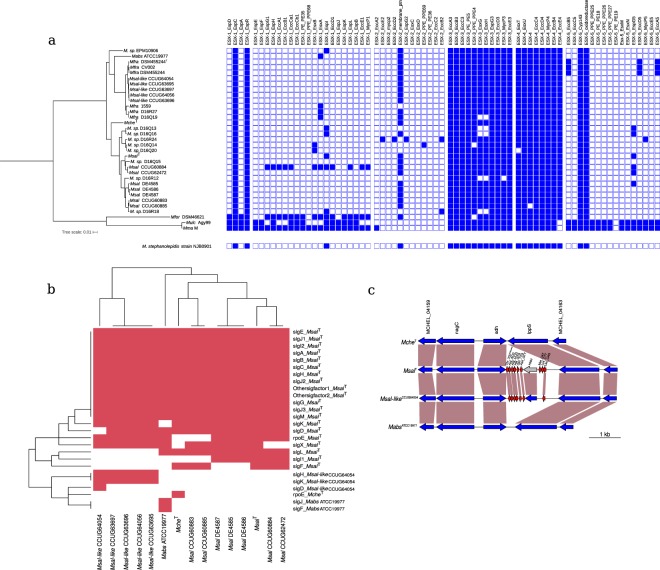
Analysis of ESX, sigma factor and tRNA genes in MCAC-members. (**a**) ESX related genes. Presence (blue) and absence (white) of ESX related genes in different mycobacteria as indicated in the phylogenetic tree shown to left (see also Fig. 4b). ESX related genes present in *M. stephanolepidis* NJB0901 is shown below, see main text. (**b**) Sigma factor genes. Heat map showing presence (red) and absence (white) of sigma factor genes in *Mabs*^ATCC19977^, *Mche*^T^, *Msal*^T^ and *Msal*-like^CCUG64054^. The signature for the respective sigma factor genes correlate with the naming for *Mtb*H37Rv sigma factor genes^43^. The horizontal and vertical trees represent the heat map clustering of the column and row wise dendograms. (**c**) Predicted presence of additional tRNA genes in MCAC-members. Gene synteny for a tRNA gene cluster encompassing nine genes in *Msal*^T^ (seven in *Mfra*^CCUG64054^). The tRNA genes are marked in red and the vertical boxes marked in brown highlight homologous genes. Note the presence of the HNH endonuclease gene (marked in gray) located within tRNA gene clusters (see main text). See also Figs S1a and S9a.

Several MCAC-members cause disease and strains belonging to the *Mabs* branch is of particular interest (see introduction). *Mfra* (including *Msal*-like, see above) is phylogenetically close to *Mabs*, and *Msal*-like strains were isolated from the water system at BMC (Uppsala University) after an outbreak in the animal facility. Hence, we were interested in to survey the presence of genes encoding for virulence factors (VF) in the *Msal* and *Msal*-like strains. For this purpose, we extracted 326 (including homologs) VF genes from a selected number of mycobacteria, including *Mtb*H37Rv (Table S8a), from the virulence factor database (VFDB; last accessed Aug 2017) and searched for orthologs in the *Msal* and *Msal*-like genomes. The presence (and absence) of VF genes in the *Msal* and *Msal*-like strains were similar compared to *Mabs*^ATCC19977^ and *Mche*^T^. Of 326 VF genes, 53 are common to all selected mycobacterial species and orthologs to roughly 40% of the 326 VF genes were predicted to be present in *Mche*^T^*, Mabs*^ATCC19977^, and in the *Msal* and *Msal*-like strains (Fig. S8; Table S8b). A comparison of *Msal* and *Msal*-like strains revealed that certain VF genes are unique to *Msal* such as *fadE*14 and *fadD*33 (also known as *mbtM* and *mbtN*, which are involved in mycobactin biosynthesis^32^). Of note, *fadE*14 and *fadD*33 are also absent in *Mabs*^ATCC19977^ (Fig. S8; Table S8b) while *Msal*-like strains have two *mbtE* orthologs, which might influence mycobactin synthesis. Moreover, *sigL* is absent in the *Msal*-like strains while *Mabs*^ATCC19977^ carries one *sigL* copy (Fig. S8; Table S8b; see below, Fig. 6b).

Two *Mabs* VFs, *adhD* and *plcC*, were predicted to be present in the *Msal*-like strains (Table S8b). The *adhD* encodes a potential zinc-type alcohol dehydrogenase, while *plcC* encodes phospholipase C that hydrolyses membrane phospholipids. The presence of *plcC* makes *Mabs* highly cytotoxic to mouse macrophages and, as such *plcC* is a major virulence factor in *Mabs*^33^.

In general, MCAC-members lack ESX-1, ESX-2 and ESX-5 genes (Fig. 6a), where ESX-1 (which encode for *esxA/*ESAT-6 and *esxB*/Cfp-10) and ESX-5 have an impact on mycobacterial virulence^34,35^. However, a few ESX-1, ESX-2 and ESX-5 homologs were predicted to be present in some of the species, where *Msal*^CCUG60884^ encodes for several ESX-1 genes, including *esxA* and *esxB*. We also noted that *Mycobacterium stephanolepidis* NJB0901 lacks ESX-1, ESX-2 and ESX-5 genes as other MCAC-members (Fig. 6a; see also^36^). Moreover, homologs of the VF gene *espC* were predicted to be present in the MCAC-members (Fig. 6a). The EspC protein is localized on the *Mtb* surface and is co-secreted with EsxA and EsxB^37^. In contrast to ESX-1, -2 and -5 genes, ESX-3 and ESX-4 genes were predicted to be present^38,39^ where ESX-4 is considered to be the ancestor of the ESX-systems^38^. ESX-3 is suggested to be required for mycobactin mediated iron uptake and, as such, have an impact on virulence of *Mtb*^40^ while *Mabs* ESX-4 genes contribute to intracellular survival^39^. Of note, absence of ESX-3 and ESX-4 genes is possibly due to draft genome status. Moreover, *mce* genes affect virulence^41^ and albeit MCAC-members encode several *mce* genes belonging to *mce*4 and *mce*9 they lack *mce*2, *mce*3, *mce*7 and *mce*8 genes (Fig. S8; Table S7). We also note that none of the MCAC-members carry *ctpV*, a putative copper exporter and required for full *Mtb*H37Rv virulence^42^. Together these data imply variation and differences in genes having a role for a successful infection caused by SGM such as *Mtb* and RGM MCAC-members, *e.g*. *Msal* and *Msal*-like.

### Transcription sigma factor genes

In bacteria, initiation of transcription requires sigma factors and, as such they have key roles in regulating gene expression^43–46^. While *Mtb*H37Rv is equipped with 13 different sigma factors the number varies between 17 and 19 in MCAC-members (Fig. 6b). Collectively, orthologs for almost all *Mtb*H37Rv sigma factor genes (*sigA-M*) were also predicted to be present in MCAC-members. In addition to that *sigL* appears to be missing in the *Msal*-like strains we note the following. The sigma factor C (*sigC*), which is suggested to have a role for *Mtb* virulence^47^, is present in these mycobacteria, while it is absent in the RGM *Msmeg*MC^2^155^46^. No *sigF* orthologs could be detected in the *Msal*-like strains and in *Msal*^DE4585^, *Msal*^DE4586^, and *Msal*^DE4587^. An ortholog to the *Mtb sigI* gene was predicted to be present in the *Msal* strains but it is missing in all the other strains. For *sigJ*, we predicted three orthologs in *Mche*^T^, the *Msal* and *Msal*-like strains, and four in *Mabs*^ATCC19977^ (of note, for other mycobacteria it has been reported that the *sigJ* transcript level increases in late stationary phase and during intracellular growth^48,49^). Moreover, the *Msal*-like strains carry two *sigK* orthologs, the *Msal* strains and *Mche*^T^ were predicted to have one, while we were unable to detect any *sigK* gene in *Mabs*^ATCC19977^. Interestingly, in *Mtb* SigK influences expression of the MPT70 and MPT80 antigens and it has been inferred that the SigK/anti-SigK regulatory system is conserved among mycobacteria^50^. Hence, it seems that *Mabs*^ATCC19977^ consititutes an exception. Together this suggested variation in sigma factor occurrence within MCAC with probable consequences in gene expression patterns in these species.

### Genes related to translation

Prediction of ribosomal protein (RP) genes revealed that *Msal*^T^ encodes 38 large subunit RPs, L1-L36, with two genes encoding L28, L31 and L33, respectively (Table S9a). These genes are also present in *Mche*^T^, *Mabs*^ATCC19977^ and *Msal*-like^CCUG64054^, albeit with some variations; two copies of the L30 gene is present in *Msal*-like^CCUG64054^, while the L36 gene was not detected in *Mabs*^ATCC19977^ and *Msal*-like^CCUG64054^. With respect to small subunit RPs, 23 genes were predicted, S1-S20 with two paralogs encoding for S1, S14 and S18 in *Msal*^T^, *Mche*^T^, *Mabs*^ATCC19977^, and *Msal*-like^CCUG64054^, while two S5 paralogs were also detected in *Msal*-like^CCUG64054^ (Table S9). Compared to *Mtb*H37Rv, we noted some differences; *Mtb*H37Rv encodes three L28 genes, and it lacks second copies of the L31, L33, and S1. As *Msal*^T^, *Mche*^T^, *Mabs*^ATCC19977^, *Mtb*H37Rv lacks the L36 gene and it does not carry an extra copy of the S5 gene. The presence of extra RP paralogs has been discussed to play a role in adaption to stress and for S18 data suggest that it has a role in zinc homeostasis in *Mtb*H37Rv^51,52^.

All MCAC-members encode for a complete set of translation factor genes with the exception of *prfC* (release factor 3; Table S9b). This is the case also for *Mtb*H37Rv and other SGM, while RGM such as *Msmeg*MC^2^155 and *Mycobacterium phlei* have *prfC* homologs^20,53,54^. Moreover, while *Mtb*H37Rv carries two *fusA* genes, *fusA*1 (EF-G) and *fusA*2 (extra EF-G), *Msal*^T^ and MCAC-members encode for only one *fusA* gene corresponding to *fusA*1 in *Mtb*H37Rv.

### Variations in the number of tRNA genes

MCAC-members carry between 47 and 80 tRNA genes and some also encode for pseudo tRNAs, *e.g*., *Msal*^DE4585^ and *Msal*^D16Q15^ carry two and three, respectively (Fig. 6c, S1 and S9a–f). Among the tRNA genes, 38 (“core tRNAs”) are present in all strains covering all amino acids except SelCys for which no tRNA gene could be identified. For *Mabs*^ATCC19977^, *Mche*^T^, *Msal*^T^ and *Msal*-like^CCUG64054^ the number of tRNA genes vary with 47 for the two formers and 56, and 53 for *Msal*^T^ and *Msal*-like^CCUG64054^, respectively (Fig. S9a). Their locations on the chromosome in these species are similar (Fig. S9b–d). Several of the extra tRNA genes in *Msal*^T^ cluster together and were predicted to be present in several *Msal* strains belonging in particular to *Msal* group I and II, while those present in *Msal*-like^CCUG64054^ (and *Mfra* strains; all draft genomes) likely cluster at roughly the same location on the chromosome as in *Msal*^T^ (Fig. 6c; Fig. S9a,e). Sequence alignments suggest that these extra tRNA genes probably are of different origins (Fig. S9f). Interestingly, the group III *Msal* strain, *Msp*^D16Q14^ was predicted to carry 80 tRNA genes where 34 appears to cluster (Fig. S9a). This tRNA gene cluster shows striking similarities, including the presence of the GOLLD RNA gene and an HNH endonuclease gene (not shown), with that detected in *e.g*., *Mabs* M24 and the *Mycobacterium aubagnense* type strain^55,56^ (Behra *et al*. unpublished).

To conclude, based on that the extra tRNA show differences in their structure compared to the common tRNAs it is conceivable that they have been acquired through horizontal gene transfer after divergence of *Msal* and *Mche*, and *Mabs* and *Msal*-like strains (and *Mfra*). In this context, we note that an HNH endonuclease gene is predicted to be present in close proximity to the extra tRNA gene clusters (Fig. S9e). Moreover, given that MCAC-members are the closest mycobacteria to the mycobacterial ancestor^17,18^ (unpublished) our data suggest that at least 38 tRNA genes were present before mycobacteria diverged into separate species that constitute the genus.

## Discussion

We present the genomes for 14 mycobacteria, including the complete genomes for *Msal*^T^ and *Mche*^T^ (type strains), belonging to MCAC. The size of the genomes range between 4.8 and 5.2 Mbp with *Msal*^T^ having the smallest genome. Our comparative genomic analysis, ANI, CDS amino-acid percentage identity and core gene based phylogeny, suggested that *Msal* and *Msal*-like strains are representatives of different species and close to *Mabs*. Including *Mfra*^10,12^, *Mycobacterium* sp. “D16” strains and *Mabs*^ATCC19977^ suggest that the *Msal*-like isolates should be referred to as *Mfra* strains (Fig. 4). The data further suggested that the *Msal* strains clustered into three separate groups, where the *Mycobacterium* sp. D16 (Q13, Q14, Q16, Q20 and R24) constitute one group. Together, our findings expand and provide insight into the phylogenetic and evolutionary relationships within the MCAC and the *Mycobacterium* genus and clarify species identity^17–20,57^.

The number and type of IS elements in *Msal*, *Msal*-like, *Mfra*, *Mche*^T^ and *Mabs*^ATCC19977^ genomes vary as does the presence of phage sequences, while sequences of plasmid origins were only detected in *Msal*^T^. Thus, IS elements and phages appear to have contributed to the evolution of these MCAC members. Differences with respect to IS elements and phage sequences are also observed comparing strains of other mycobacteria, as exemplified by our comparative genomic studies of *M. phlei* and *Mma* strains^20,57^. Moreover, the number of SNVs for the *Msal* strains ranged between 136702 and 291755. This is significantly higher compared to the situation in *Mma* where the number of SNVs in different strains relative to the *Mma* M strain varies between 56000 and 89000^57^. But, we emphasize that *Mma* display a higher frequency of mutational hotspot regions relative to *Msal* (26.5/Mb *vs*. 14.7/Mb, respectively). In this context, some mycobacteria use Distributive Conjugal Transfer (DCT) to transfer DNA and ESX-1 and ESX-4 have been suggested to play a key role in this process^58–60^. Analysis of *Mabs* isolates implicates that DCT is in operation in this RGM^61^. Together this makes it plausible that *Msal* strain variation and clustering into three groups is at least partly the result of DCT.

As other members of MCAC, *Msal* and *Mfra* (*Msal*-like) lack the majority of the ESX-1, ESX-2, ESX-5 and ESX-6 (duplication of ESX-1 in *Mma*^26,57^) genes, while genes belonging to ESX-3 and ESX-4 are present. However, some ESX genes such as *esxA*, *esxB* and *espI* (associated with ESX-1) were predicted to be present in some of the strains, while the ESX-1 associated *espR* is present in all strains (see below). Interestingly, *Msal*^CCUG60884^ encodes for several of the ESX-1 genes including *esxA* and *esxB* (Fig. 6a). Possibly, these ESX genes have been aquired through horizontal gene transfer. The ESX systems are involved in transport and secretion and available data suggest that ESX-1, ESX-3 and ESX-5 affect virulence for several mycobacteria including *Mtb* and *Mma*^34,35^. That ESX-5 is missing is consistent with that it is present in SGM and has not been detected in RGM^35^ (unpublished data).

The transcriptional regulator EspR (*espR*) is involved in controlling *Mtb* virulence and expression of ESX-1 genes^62^ and *espR* is present in MCAC-members. Apart from being a regulator of the ESX-1 system, EspR is regulating the expression of genes involved in cell wall synthesis. EspR also operates together with PhoP, which is part of a two-component system, regulating the expression of many VF genes. This provides a rationale for its presence in MCAC-members and given that *espR* constitutes a hotspot region in *Msal* raises the possibility that this has an impact on the pathogenicity for the different *Msal* strains. In this context, we note that *espR* is not essential in *Mtb*H37Rv^62^.

An intriguing question is whether other genes/systems functionally compensate for the absence of these ESX systems. For example, there appears to be a coupling between the transporter Mce1 family proteins and the ESX-1 system^35^. Understanding this and other questions that relates to mycobacterial infections will have an impact on our understanding of the biology of mycobacteria. In this context, the PhoPR regulon and ESX-1 secretion in a *Mtb*CDC1551 derivative is inhibited by ethoxzolamide, which is a known carbonic anhydrase inhibitor. As a consequence, virulence is attenuated. Together, this raises the possibility of coupling between carbonic anhydrase activity and signaling mediated by the two-component PhoPR regulatory system in *Mtb*CDC1551^63^. A carbonic anhydrase 1 gene was predicted to be present in *Mabs*^ATCC19977^ and *Msal*-like^CCUG64054^ but absent in *Msal*^T^ and *Mche*^T^. Hence, it would be interesting to study whether this coupling is present in *Mabs* (and *Msal*-like *i.e*., *Mfra*) and if so, does ethoxzolamide also influence the virulence for these two mycobacteria.

As other MCAC-members, *Msal* and *Msal*-like belong to RGM but they only harbor one rRNA operon supporting the notion that the number of rRNA operons does not explain the difference in growth rate comparing SGM and RGM^57,64–66^. Moreover, as *Mtb* and other SGM, *Msal*^T^ (complete genome) and other MCAC-members lack the gene encoding the translational release factor RF3, *prfC*. RF3 is suggested to assist in the dissociation of class I translational release factors from the ribosome, and to abolish competition between the release factors and ribosome recycling factor, RRF, for binding to the ribosome^67–69^. Inactivation of RF3 in *Escherichia coli* results in lower growth rates^67,70,71^. Together this suggests that the absence or presence of *prfC* does not dictate whether mycobacteria should be classified as SGM or RGM. Moreover, phylogeny based on whole genome sequencing suggests that MCAC is the earliest diverging mycobacterial lineage^17,18,36^ (unpublished). Hence, acquisition of *prfC* in other RGM happened after they diverged from MCAC.

MCAC-members, except *Msal*, have been implicated to be associated with human diseases. *Mfra*, which belongs to MCAC, causes symptoms similar to those observed in patients infected with *Mabs*^10,12^. We cannot conclusively state that the *Msal*-like strains isolated from the tap water caused the outbreak at the animal facility among the mice population at Uppsala University. To do this the pathogen has to be isolated from infected mice and study whether exposure to the bacteria indeed cause disease. However, on the basis that *Mfra* causes disease in humans we consider it plausible that *Msal*-like (*i.e*. *Mfra*) also infects and causes disease in animals, such as mice. In this context, *Msal*-like (and *Mfra*) strains share several genes coding for virulence factors (VF) with *Mabs* such as the major *Mabs* VF *plcC*, which makes *Mabs* highly cytotoxic to mouse macrophages^33^. Nonetheless, its presence in tap water suggest that it can be present in the environment. Moreover, we note that neither of the strains analyzed here carry mutations at positions 1408 (16S rDNA; *E. coli* numbering) or 2058 (or 2059; 23S rDNA). Mutations at these positions in *Mabs* isolates results in resistance to amikacin and macrolides, respectively^72^.

To conclude, understanding the genome composition of mycobacteria will be instrumental to understand not only their evolution but also provide insight into mycobacterial physiology and pathogenicity, and clarify species identity. This knowledge will be instrumental for treatment of infections caused by mycobacteria such as MCAC-members.

## Methods

### Strains, cultivation and DNA isolation

We collected eight *Msal* and five *Msal-*like strains isolated from different sources, *Mfra*^DSM45524T^ and *Mche*^T^ where *Msal*^T^ and *Mche*^T^ represent the type strains *Msal* DSM43276 and *Mche* DSM43804, respectively. *Msal*^T^, *Mche*^T^ and *Mfra*^DSM45524T^ were obtained from the Deutsche Sammlung von Mikrooganismen und Zellkulturen, Germany (Table 1 and S1; we refer to the strains such that *e.g*., strain *Msal*^DE4585^ corresponds to *M. salmoniphilum* DE4585). The strains were grown under conditions as recommended by the supplier (for the outbreak *Msal* “DE-strains”, we followed the recommendation from DSM); aliquots of −80 °C stocks were plated on Middlebrook 7H10 media and incubated at 30 °C. Genomic DNA was isolated as previously described^73^ (see also Supplementary information). Prior to submission for genome sequencing we PCR amplified and sequenced 16S rDNA to ensure that the cultures were free from contaminations.

### Genome sequencing, assembly and annotation

The *Msal*^T^ and *Mche*^T^ type strains were sequenced using the Pacific Biosciences (PacBio) platform at the NGI-Uppsala Genome Center, while sequencing of the other 12 strains (and *Mfra*^DSM45524T^) were performed using Illumnia short read technology (at the SNP@SEQ Technology Platform, Uppsala University). Genome assembly, annotation, plasmid, phage, identification of IS elements, horizontal gene transfer (HGT) analysis and identification of SNV and mutational hotspots were done as previously described^19,20,30,57^ (see Supplementary information and refs^74–83^).

### Average nucleotide identity

The evolutionary distance between two species can be measured as average nucleotide identity (ANI) of homologous genomic regions^84^. ANI values were calculated for all the sequenced genomes in a pairwise manner using the Jspecies tool^21^. The ANI values were clustered using an unsupervised hierarchical clustering algorithm and plotted using “R” environment^85^.

### Identification and analysis of core genes

To identify core and unique genes, predicted CDS from the genomes were used for “all-*vs*-all” BLAST search. Based on the BLAST results orthologous genes were identified using PanOCT with minimum 45% identity and 65% query coverage^86^, see also refs^19,20,57^.

### Phylogenetic analysis based on single and multiple genes

We extracted 16S ribosomal RNA (rRNA) gene sequences from the genomes and homologous gene sequences from other mycobacteria as indicated were downloaded from the NCBI database and aligned using MAFFT (version 5^87^). Phylogenetic trees, 16S rDNA and core gene based trees, based on the multiple sequence alignment were computed using the FastTree along with default settings, which infers approximately-maximum-likelihood phylogenetic trees from alignments of nucleotide or protein sequences (Jukes-Cantor + CAT model for nucleotide sequences and Jones-Taylor-Thorton + CAT models of amino acid sequences) and 1000 cycles of bootstrapping^88^. The figures were generated using FigTree (http://tree.bio.ed.ac.uk/software/figtree/).

### Ethics statement

All methods were carried out in accordance with relevant guidelines and regulations.

### Data deposition

This Whole Genome Shotgun project has been deposited at DDBJ/ENA/GenBank under the projects PRJNA414709, PRJNA508902 and PRJNA509866.

## Supplementary information

Supplementary Information

Supplementary Table S1

Supplementary Table S2

Supplementary Table S3

Supplementary Table S4

Supplementary Table S5a-e

Supplementary Table S6

Supplementary Table S7

Supplementary Table S8a, b

Supplementary Table S9
