# Supplementary material for: Extended insight into the Mycobacterium chelonae-abscessus complex through whole genome sequencing of Mycobacterium salmoniphilum outbreak and Mycobacterium salmoniphilum-like strains
Source: Sci Rep. 2019 Mar 14;9:4603. doi: 10.1038/s41598-019-40922-x (PMC6418233; doi:10.1038/s41598-019-40922-x)

Supplementary information

Extended insight into the *Mycobacterium chelonae-abscessus* complex through whole genome sequencing of *Mycobacterium salmoniphilum* outbreak and *Mycobacterium salmoniphilum*-like strains

Phani Rama Krishna Behra[1#], Sarbashis Das[1#], B. M. Fredrik Pettersson[1], Lisa Shirreff[2], Tanner DuCote[2], Karl-Gustav Jacobsson[3], Don G. Ennis[2] and Leif A. Kirsebom[1*]

[1]Department of Cell and Molecular Biology
Box 596, Biomedical Centre
SE-751 24 Uppsala, Sweden

[2]Department of Biology,
University of Louisiana,
Lafayette, Louisiana, USA

[3]Department of Neuroscience
Box 593, Biomedical Centre
SE-751 24 Uppsala, Sweden

[#]These authors contributed equally to this work

*corresponding author
Leif.kirsebom@icm.uu.se

Supplementary Methods: DNA isolation, Genome assembly, annotation, plasmid, phage, identification of IS elements, horizontal gene transfer (HGT) analysis and identification of SNV and mutational hotspots.

Supplementary Table S1: Compilation of mycobacterial species/strains, and genomes used in the present study.

Supplementary Table S2: Summary of predicted ncRNA genes in MCAC members.

Supplementary Table S3: Summary of predicted phages in the different MCAC members.

Supplementary Table S4: Summary of IS-elemnets in the different MCAC members.

Supplementary Table S5a-e: List of core and unique genes in *Msal*^{T}, *Mche*^{T}, *Msal*-like^{CCUG64054} and *Mabs*^{ATCC19977}.

Supplementary Table S6: List of hotspot genes, annotation and function.

Supplementary Table S7: Horizontal gene transfer analysis. Sheet 1, summary of predicted HGT genes in *Msal* and *Msal*-like strains and other mycobacteria. Subsequent sheets contain detail information of predicted HGT genes in individual strains.

Supplementary Table S8: Virulence factor analysis. Sheet 1, list of genomes used in VF analysis and sheet 2 list predicted virulence genes along with functional classification for *Msal* and *Msal*-like strains and other mycobacteria.

Supplementary Table S9a, b: (a) List of genes encoding ribosomal proteins in MCAC-members. (b) List of genes encoding translation factors in MCAC-members.

Supplementary Methods

DNA isolation

For Illumina sequencing, cells were lyzed by bead beating (2 x 1 min, 6.5 m/s, 5 min on ice between runs, 0.1 mm silica/zirconium beads) in equal volumes of TE-buffer (10 mM Tris-HCl, pH 7.5; 1 mM EDTA) and DNAZol reagent (Invitrogen) using a FastPrep24 device (MP Biotech). This was followed by chloroform extraction and ethanol precipitation, resuspension in 1 x TE-buffer and removal of RNA and proteins using RNase A and Proteinase K treatment for 1 h each. Chromosomal DNA was retrieved by phenol/chloroform extraction and ethanol precipitation.

For PacBio sequencing, 500 mL of exponentially growing culture was pelleted and resuspended in 11 mL of Qiagen buffer B1 (containing 1 mg/mL RNase A) and transferred to a tube containing 2 g (≥ 60000 U) Lipase (product number 80612, Sigma-Aldrich). After dissolving the Lipase, the tubes were incubated for 2 h at 37°C in a waterbath followed by: addition of 600 µL lysozyme (100 mg/mL) and 3 h of incubation, addition of 500 µL of Proteinase K (20 mg/mL) and incubation for 1.5 h, and addition of Qiagen buffer B2 and incubation for 16 h at 50°C. The cell lysate was cleared by centrifugation. The DNA was recovered using Qiagen Genomic-tip 500/G following the protocol supplied by the manufacturer and further purified using the MoBio PowerClean Pro DNA Clean-Up Kit.

Before submitting DNA for sequencing (PacBio or Illumina), DNA size and quality was estimated using spectrophotometry and agarose gel electrophoresis.

Genome assembly, annotation, plasmid, phage, identification of IS elements, identification of SNV and mutational hotspots and horizontal gene transfer (HGT) analysis

Genome assembly: The PacBio-generated reads were assembled using the SMRT-analysis HGAP3 assembly pipeline (Chin *et al*. 2013) and polished using Quiver (Pacific Biosciences,

Menlo Park, CA, USA). Assembly of the Illumina generated reads was performed using the A5-Assembly pipeline (version 20140604) with a minimum contig size of 200 bases (Tritt *et al.* 2012). The MAUVE program (Darling et al. 2004) was used for genome reordering and whole genome alignment. This alignment was plotted using genoplotR (Guy et al. 2010). The RNAmmer (Lagesen *et al.* 2007) and tRNAScan-SE (Lowe and Eddy 1997) programs were used to predict the rRNA and tRNA genes. All the genomes were annotated and functionally classified using Prokka [version 1.11] (Seemann 2014) and RAST server (http://rast.nmpdr.org/) (Aziz *et al.* 2008), respectively.

Plasmid, phage and IS element predictions: Assembled scaffolds were subjected to BLAST search using the NCBI plasmid database (downloaded March 2016). We considered a scaffold belonging to a plasmid if more than 90% of the scaffold sequence aligned with a plasmid sequence from the plasmid database with more than 90% identity.

Phage sequences were predicted using the PHAST server (Zhou *et al.* 2011), while prediction of IS elements was done using the ISsaga webserver (Varani *et al.* 2011).

Identifications of mutational hotspots and SNVs: Mutational hotspots were identified using Shewhart Control Chart, as described by Das *et al.* (2012). Briefly, SNVs were identified between *Msal*^{T} and other *Msal* strains in a pairwise manner using MUMmer (Delcher *et al.* 1999). The reference genome *Msal*^{T} was divided into non-overlapping windows of 2000 bases and the average number of SNVs in each of the windows was determined. The average SNV values were subsequently used in Shewhart Control Chart for the prediction of hotspots.

Horizontal Gene Transfer (*HGT*): Putative horizontal gene transfer events were predicted using the tool HGTector v0.2.2 (Zhu *et al.* 2014). Briefly, this approach is a combination of BLASTp and taxonomy searches. For the BLASTp search analysis, we used the DIAMOND v0.9.10 tool and build the database file (NCBI NR database downloaded from the HGTector source v2017-6-30) (Buchfink et al. 2015). The parameters we used for the BLASTp analysis,

percentage identity = >60% and query coverage = >70%, e-value = <1e-100. For the taxonomy search (using HGTector), we used three parameters, "self" group, "close" group, and "distal" group hierarchical classification to predict putative horizontal genes where "self = Mycobacteriaceae", and "close = Corynebacteriales" (as of Feb 2018, NCBI taxonomy; Sayers *et al*. 2009). The "distal" group = all other organisms except the "self" and "close" groups (Zhu *et al*. 2014; see Supplementary Table S7 for further information). Finally, the predicted HGTs were analysed by performing a Mann–Whitney-Wilcoxon test (in R ver 3.2.2, 2015-08-14, on platform x86_64-pc-linux-gnu) for GC-content of genome-encoded protein-coding gene sequences (CDS, excluding horizontal transferred genes) and GC-content for candidate horizontal transferred genes.

To identify common and unique genes in *Msal*^{T}, *Mche*^{T}, *Msal*-like^{CCUG64054} and *Mabs*^{ATCC19977} we combined PanOCT ortholog clustering (Fouts *et al*. 2012) with BLASTp search (Boratyn *et al*. 2013) and cut-off e-value = <1e-05, percent identity = >45% and query coverage = >70%.

References

Aziz, R. K. *et al*. The RAST Server: rapid annotations using subsystems technology. *BMC Genomics* **9**, 75 (2008).

Buchfink, B., Xie, C. & Huson, D.H. Fast and sensitive protein alignment using DIAMOND. *Nat Meth* **12**, 59–60 (2015).

Boratyn, G. M. *et al*. BLAST: a more efficient report with usability improvements. *Nucl Acids Res* **41**, W29–W33 (2013).

Chin, C-S. *et al*. Nonhybrid, finished microbial genome assemblies from long-read SMRT sequencing data. *Nat Meth* **10**, 563–569 (2013).

Darling, A. C. E., Mau, B., Blattner, F. R. & Perna, N. T. Mauve: multiple alignment of conserved genomic sequence with rearrangements. *Genome Res* 14, 1394–1403 (2004).

Das, S. *et al*. Identification of hot and cold spots in genome of Mycobacterium tuberculosis using Shewhart Control Charts. *Sci Rep* **2**, 297 (2012).

Delcher, A. L., Kasif, S., Fleischmann, R. D., Peterson, J., White, O. & Salzberg, S. L. Alignment of whole genomes. *Nucl Acids Res* **27**, 2369-2376 (1999).

Fouts, D. E., Brinkac, L., Beck, E., Inman, J. & Sutton, G. PanOCT: automated clustering of orthologs using conserved gene neighborhood for pan-genomic analysis of bacterial strains and closely related species. *Nucl Acids Res* **40**, e172–e172 (2012).

Guy, L., Kultima, J. R. & Andersson, S. G. E. genoPlotR: comparative gene and genome visualization in R. *Bioinformatics* (Oxford, England) **26**, 2334–2335 (2010).

Lagesen, K. *et al*. RNAmmer: consistent and rapid annotation of ribosomal RNA genes. *Nucl Acids Res* **35**, 3100-3108 (2007).

Lowe, T. M. & Eddy, S. R. tRNAscan-SE: a program for improved detection of transfer RNA genes in genomic sequence. *Nucl Acids Res* **25**, 955-964 (1997).

Sayers, E. W. *et al*. Database resources of the National Center for Biotechnology Information. *Nucl Acids Res* **37**, D5-15 (2009).

Seemann, T. Prokka: rapid prokaryotic genome annotation. *Bioinformatics* (Oxford, England). **30**, 2068–2069 (2014).

Tritt, A., Eisen, J. A., Facciotti, M. T. & Darling, A. E. An integrated pipeline for de novo assembly of microbial genomes. *PLoS ONE*. **7**, e42304 (2012).

Varani, A.M., Siguier, P., Gourbeyre, E., Charneau, V. & Chandler, M. ISsaga is an ensemble of web-based methods for high throughput identification and semi-automatic annotation of insertion sequences in prokaryotic genomes. *Genome Biol* **12**, R30 (2011).

Zhou, Y., Liang, Y., Lynch, K. H., Dennis, J. J. & Wishart, D. S. PHAST: a fast phage search tool. *Nucl Acids Re*s **39**, W347–52 (2011).

Zhu, Q., Kosoy, M. & Dittmar, K. HGTector: an automated method facilitating genome-wide discovery of putative horizontal gene transfers. *BMC Genomics* **15**, 717 (2014).

Figure S1 Genome alignment and genome-wide distribution of tRNA genes.

(a) Whole-genome alignment for the complete genomes *Mche*^{T}, *Msal*^{T}, *Mabs*^{ATCC19977} and the *Msal*-like^{CCUG64054} draft genome. Each horizontal block represents one genome and vertical lines between the genomes correspond to homologous regions whereas blue diagonal lines correspond to genomic inversions. Of note, we cannot conclusively state that the indicated inversion in *Msal*-like^{CCUG64054} is real due to draft genome status. White gaps correspond to the absence of genes while regions in black represent phage sequences; red stars mark intact phages while black stars mark incomplete/questionable phage sequences (see text for details).

(b) *Msal*^{T} complete genome, blue and red marked tRNA genes refers to transcription from the positive and negative strands, respectively.

(c) *Mche*^{T} complete genome, tRNA genes in red and blue as in (b).

Figure-S1:

a)

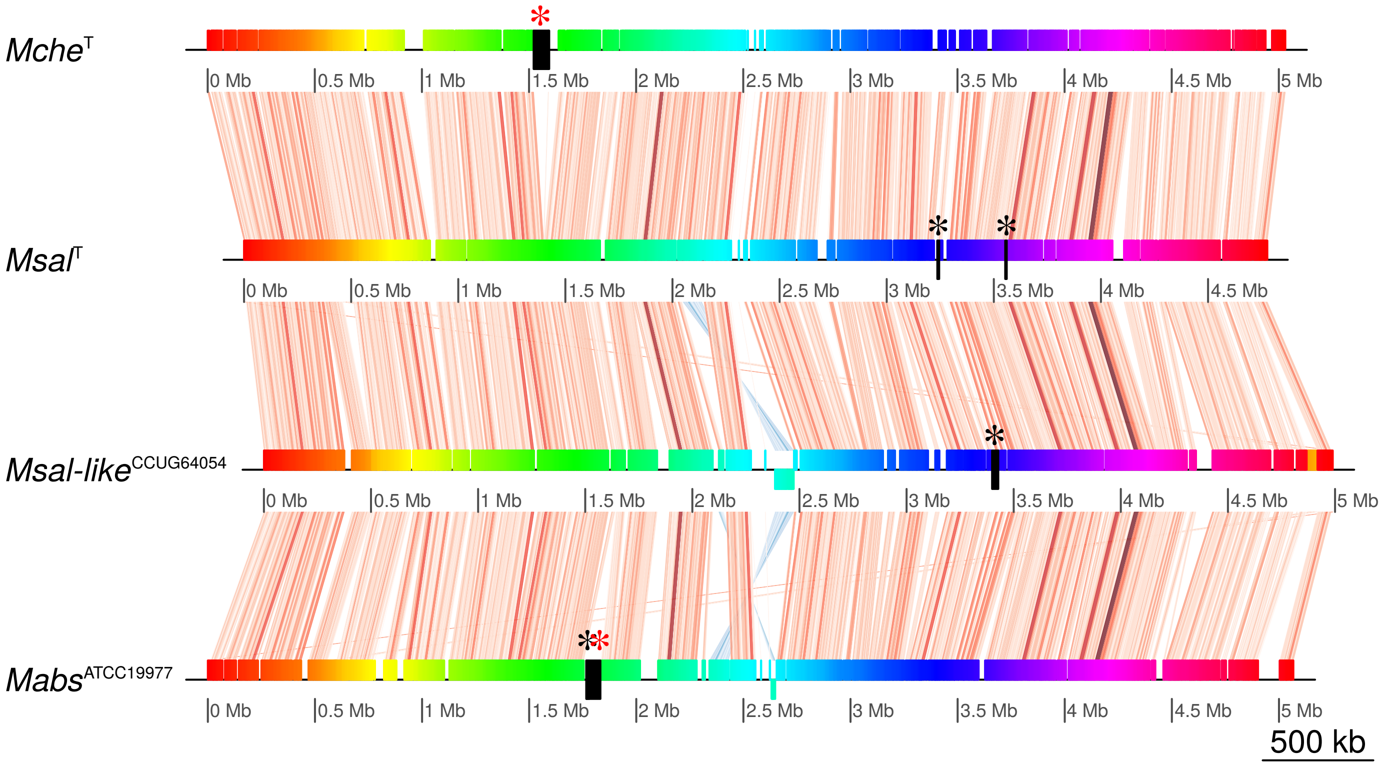

McheT
0 Mb
0.5 Mb
1 Mb
1.5 Mb
2 Mb
2.5 Mb
3 Mb
3.5 Mb
4 Mb
4.5 Mb
5 Mb
MsalT
Msal-likeCCUG64054
MabsATCC19977
500 kb

Figure-S1:

b)

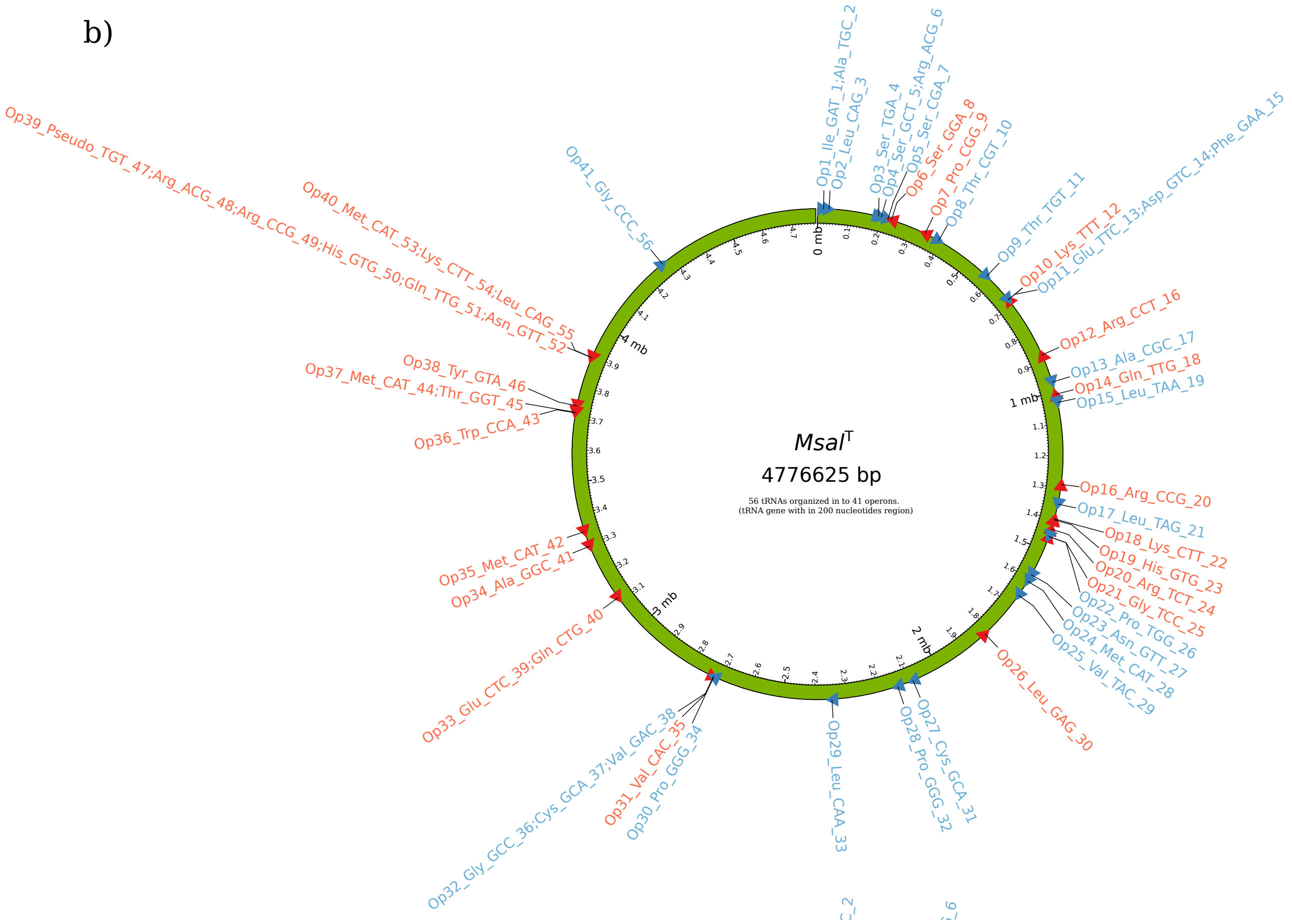

MsalT
4776625 bp
56 tRNAs organized in to 41 operons.
(tRNA gene with in 200 nucleotides region)
Op1_Ile_GAT_1;Ala_TGC_2
Op2_Leu_CAG_3
Op3_Ser_TGA_4
Op4_Ser_GCT_5;Arg_ACG_6
Op5_Ser_CGA_7
Op6_Ser_GGA_8
Op7_Pro_CGG_9
Op8_Thr_CGT_10
Op9_Thr_TGT_11
Op10_Lys_TTT_12
Op11_Glu_TTC_13;Asp_GTC_14;Phe_GAA_15
Op12_Arg_CCT_16
Op13_Ala_CGC_17
Op14_Gln_TTG_18
Op15_Leu_TAA_19
Op16_Arg_CCG_20
Op17_Leu_TAG_21
Op18_Lys_CTT_22
Op19_His_GTG_23
Op20_Arg_TCT_24
Op21_Gly_TCC_25
Op22_Pro_TGG_26
Op23_Asn_GTT_27
Op24_Met_CAT_28
Op25_Val_TAC_29
Op26_Leu_GAG_30
Op27_Cys_GCA_31
Op28_Pro_GGG_32
Op29_Leu_CAA_33
Op30_Pro_GGG_34
Op31_Val_CAC_35
Op32_Gly_GCC_36;Cys_GCA_37;Val_GAC_38
Op33_Glu_CTC_39;Gln_CTG_40
Op34_Ala_GGC_41
Op35_Met_CAT_42
Op36_Trp_CCA_43
Op37_Met_CAT_44;Thr_GGT_45
Op38_Tyr_GTA_46
Op39_Pseudo_TGT_47;Arg_ACG_48;Arg_CCG_49;His_GTG_50;Gln_TTG_51;Asn_GTT_52
Op40_Met_CAT_53;Lys_CTT_54;Leu_CAG_55
Op41_Gly_CCC_56
0 mb
1 mb
2 mb
3 mb
4 mb

c)

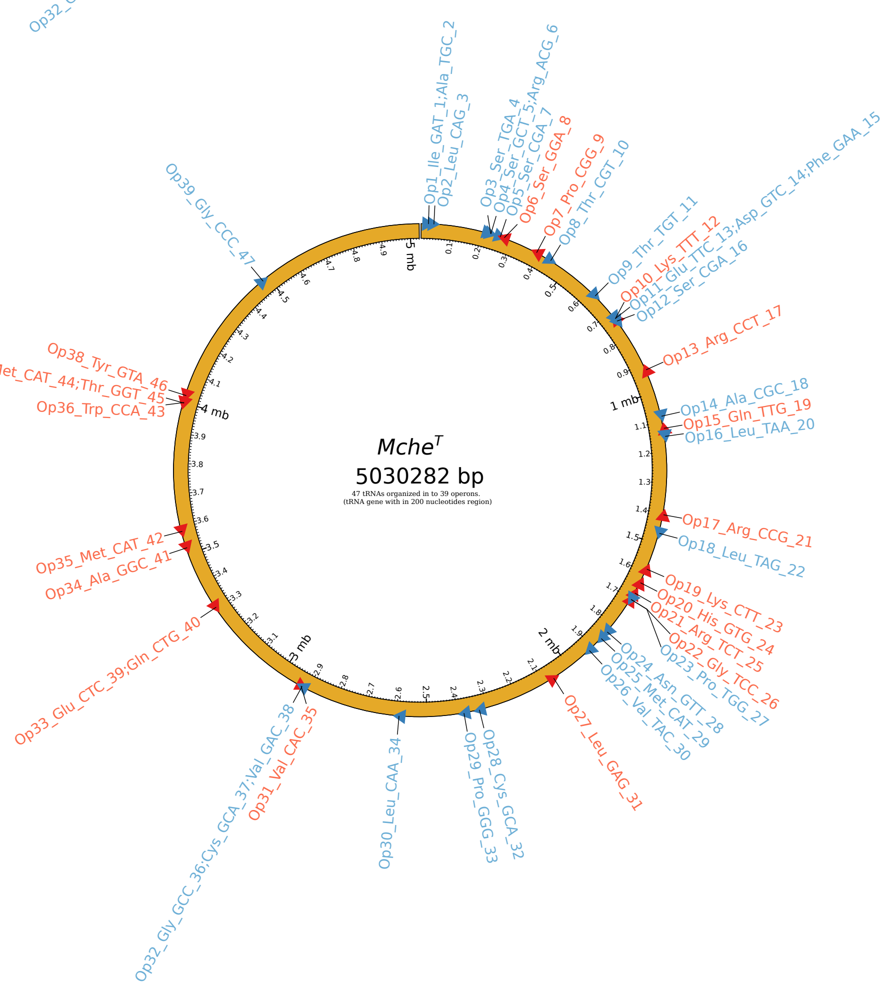

McheT
5030282 bp
47 tRNAs organized in to 39 operons.
(tRNA gene with in 200 nucleotides region)
Op1_Ile_GAT_1;Ala_TGC_2
Op2_Leu_CAG_3
Op3_Ser_TGA_4
Op4_Ser_GCT_5;Arg_ACG_6
Op5_Ser_CGA_7
Op6_Ser_GGA_8
Op7_Pro_CGG_9
Op8_Thr_CGT_10
Op9_Thr_TGT_11
Op10_Lys_TTT_12
Op11_Glu_TTC_13;Asp_GTC_14;Phe_GAA_15
Op12_Ser_CGA_16
Op13_Arg_CCT_17
Op14_Ala_CGC_18
Op15_Gln_TTG_19
Op16_Leu_TAA_20
Op17_Arg_CCG_21
Op18_Leu_TAG_22
Op19_Lys_CTT_23
Op20_His_GTG_24
Op21_Arg_TCT_25
Op22_Gly_TCC_26
Op23_Pro_TGG_27
Op24_Asn_GTT_28
Op25_Met_CAT_29
Op26_Val_TAC_30
Op27_Leu_GAG_31
Op28_Cys_GCA_32
Op29_Pro_GGG_33
Op30_Leu_CAA_34
Op31_Val_CAC_35
Op32_Gly_GCC_36;Cys_GCA_37;Val_GAC_38
Op33_Glu_CTC_39;Gln_CTG_40
Op34_Ala_GGC_41
Op35_Met_CAT_42
Op36_Trp_CCA_43
Op37_Met_CAT_44;Thr_GGT_45
Op38_Tyr_GTA_46
Op39_Gly_CCC_47
5 mb
1 mb
2 mb
3 mb
4 mb

Figure S2 Overview of genome assembly of *Msal* and *Msal*-like strains, and *Mfra*$^{\mathrm{DSM45524T}}$ (Illumina derived sequences/reads).

Figure-S2:

a.

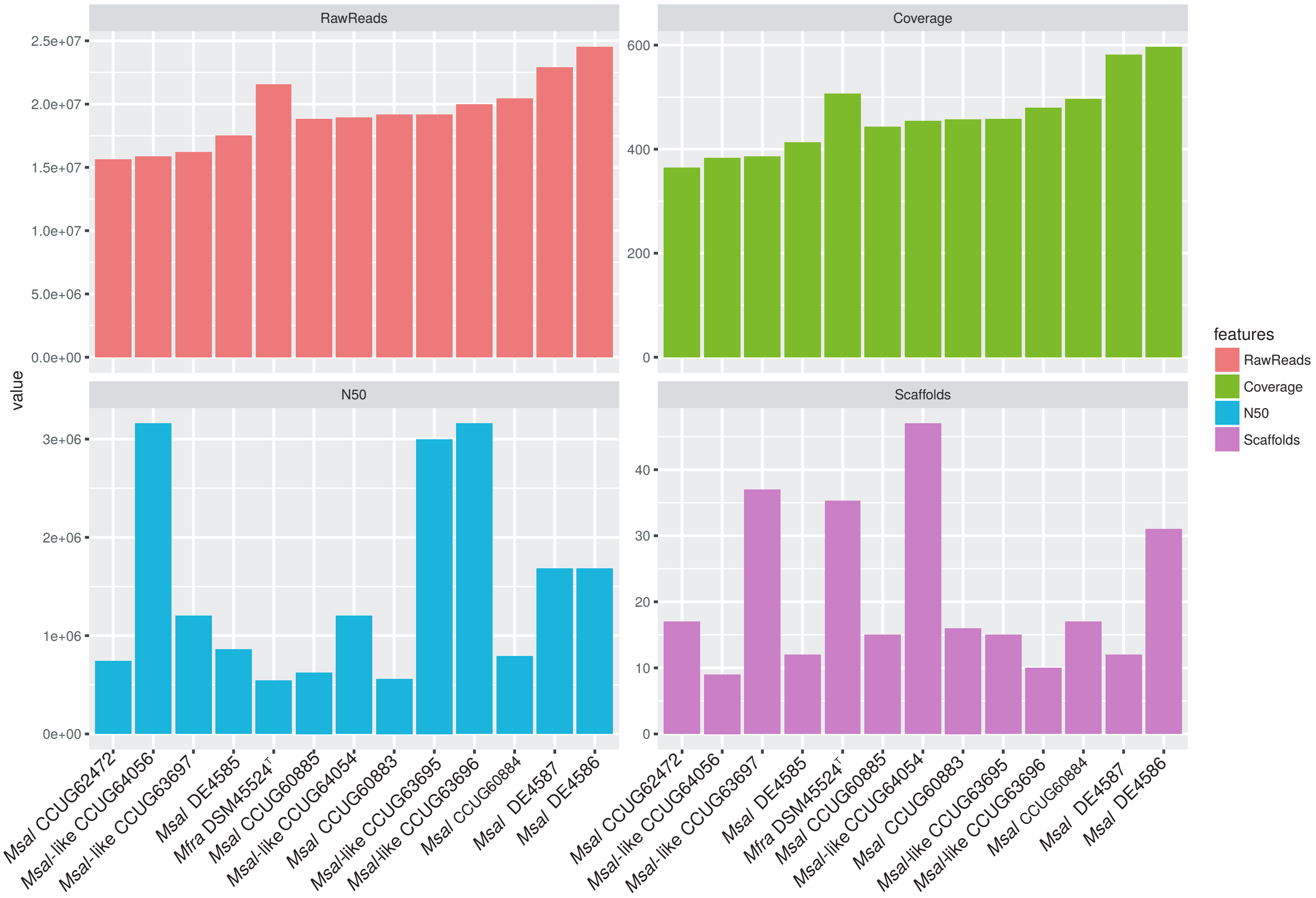

RawReads
Coverage
N50
Scaffolds
value
features
RawReads
Coverage
N50
Scaffolds
Msal CCUG62472
Msal-like CCUG64056
Msal-like CCUG63697
Msal DE4585
Mfra DSM45524T
Msal CCUG60885
Msal-like CCUG64054
Msal CCUG60883
Msal-like CCUG63695
Msal-like CCUG63696
Msal CCUG60884
Msal DE4587
Msal DE4586

Figure S3 Bar plot showing predicted cumultative phage sequence lengths and classification as indicated (see also main text). X and Y axis indicates species/strain names and length of the phage sequence in kilo bases (Kb), respectively.

Figure-S3:

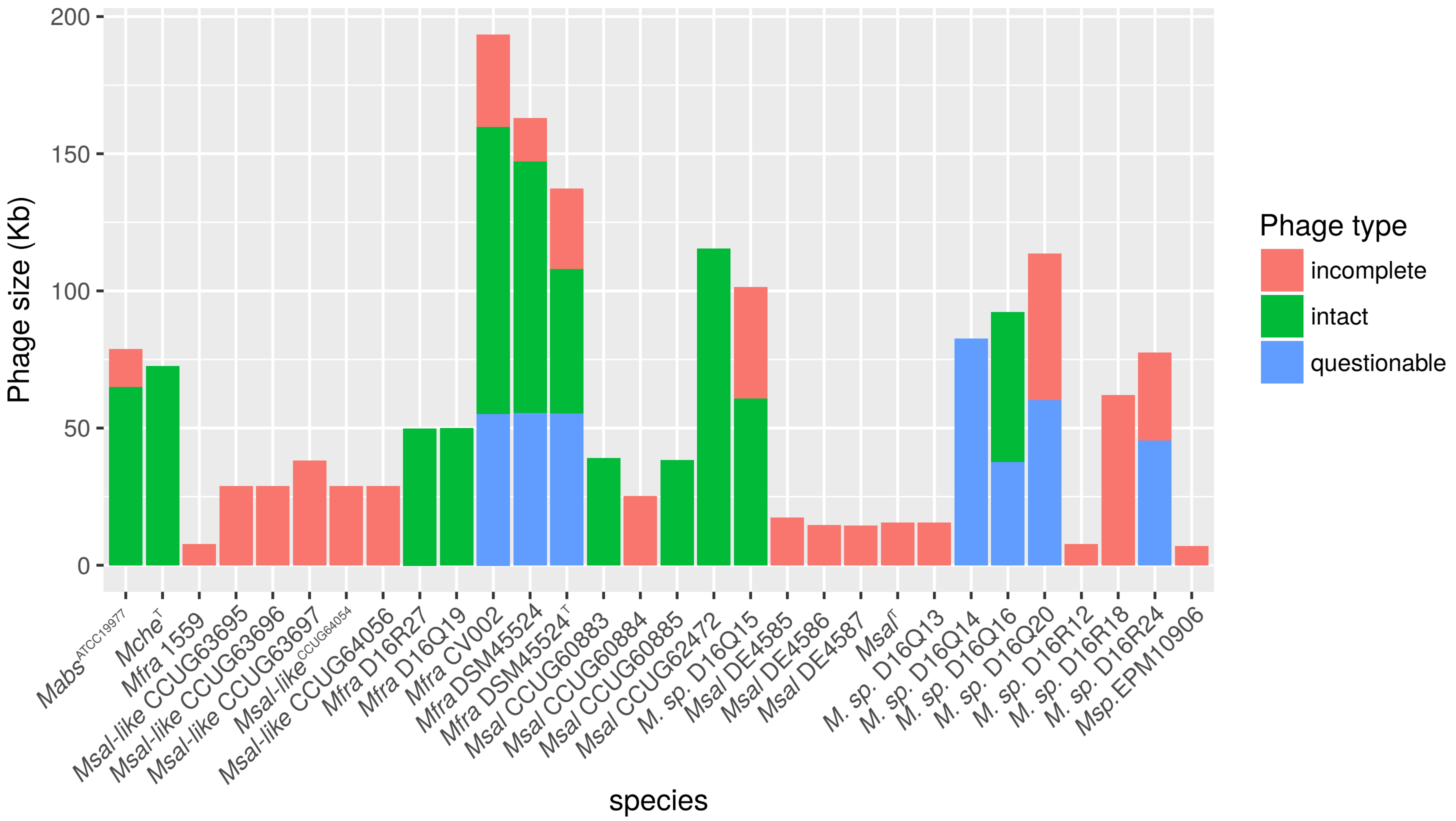
200
150
100
50
0
Phage size (Kb)
Phage type
incomplete
intact
questionable
Mabs ATCC19977
Mche T
Mfra 1559
Msal-like CCUG63695
Msal-like CCUG63696
Msal-like CCUG63697
Msal-like CCUG64054
Msal-like CCUG64056
Mfra D16R27
Mfra D16Q19
Mfra CV002
Mfra DSM45524
Mfra DSM45524 T
Msal CCUG60883
Msal CCUG60884
Msal CCUG60885
Msal CCUG62472
M. sp. D16Q15
Msal DE4585
Msal DE4586
Msal DE4587
Msal T
M. sp. D16Q13
M. sp. D16Q14
M. sp. D16Q16
M. sp. D16Q20
M. sp. D16R12
M. sp. D16R18
M. sp. D16R24
Msp.EPM10906
species

Figure S4 (a) Amino-acid percentage identity plots for the different MCAC-members shown in Fig 3a (see main text) and as indicated. Y-axis (count) refers to the number of genes while the X-axis gives the percentage identity (PID).

(b) Alignment of the *ileS* sequences upstream of the predicted translational start sites (marked ATG and GTG codons) in MCAC-members, *Mtb*H37Rv and *Msmeg*MC^{2}155 as indicated. Red and blue residues mark the "T-box" signatures (see main text for references).

(c) A secondary structure model of the *Mabs*^{ATCC19977} T-box using the *Msmeg*MC^{2}155 T-box as template (see Ref 23 main text). The highligthed boxes (dashed lines) marks K-turn and putative S-turn motifs while • mark conserved residues in the K-turn, S-turn and T-box. The inset highlight the S-turn region in *Msmeg*MC^{2}155 and the putative S-turn structure in *Msal*^{T}.

Figure-S4:

a.

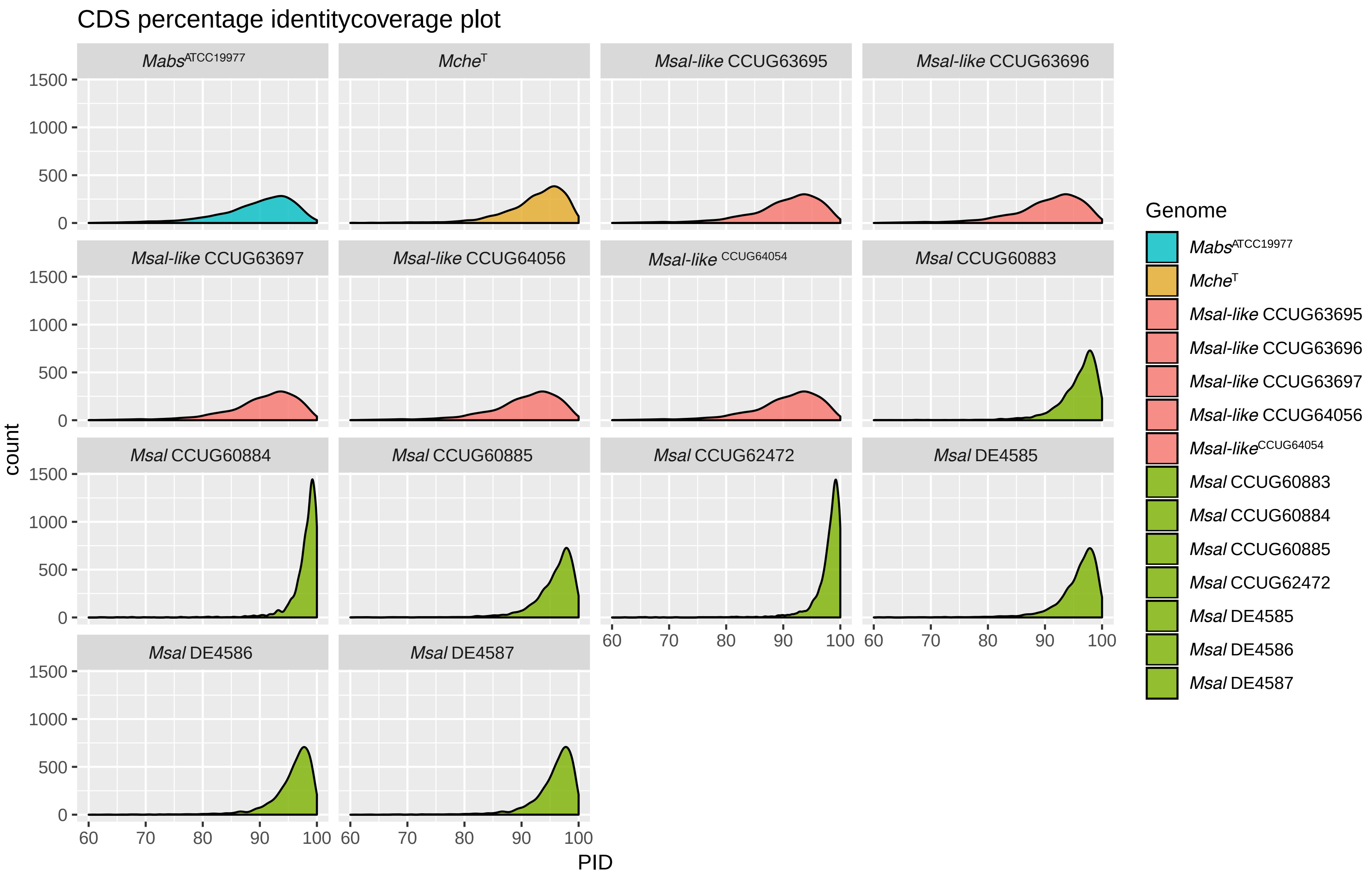

CDS percentage identitycoverage plot
MabsATCC19977
McheT
Msal-like CCUG63695
Msal-like CCUG63696
Msal-like CCUG63697
Msal-like CCUG64056
Msal-like CCUG64054
Msal CCUG60883
Msal CCUG60884
Msal CCUG60885
Msal CCUG62472
Msal DE4585
Msal DE4586
Msal DE4587
count
0
500
1000
1500
60
70
80
90
100
PID
Genome
MabsATCC19977
McheT
Msal-like CCUG63695
Msal-like CCUG63696
Msal-like CCUG63697
Msal-like CCUG64056
Msal-likeCCUG64054
Msal CCUG60883
Msal CCUG60884
Msal CCUG60885
Msal CCUG62472
Msal DE4585
Msal DE4586
Msal DE4587

Figure-S4:

b.

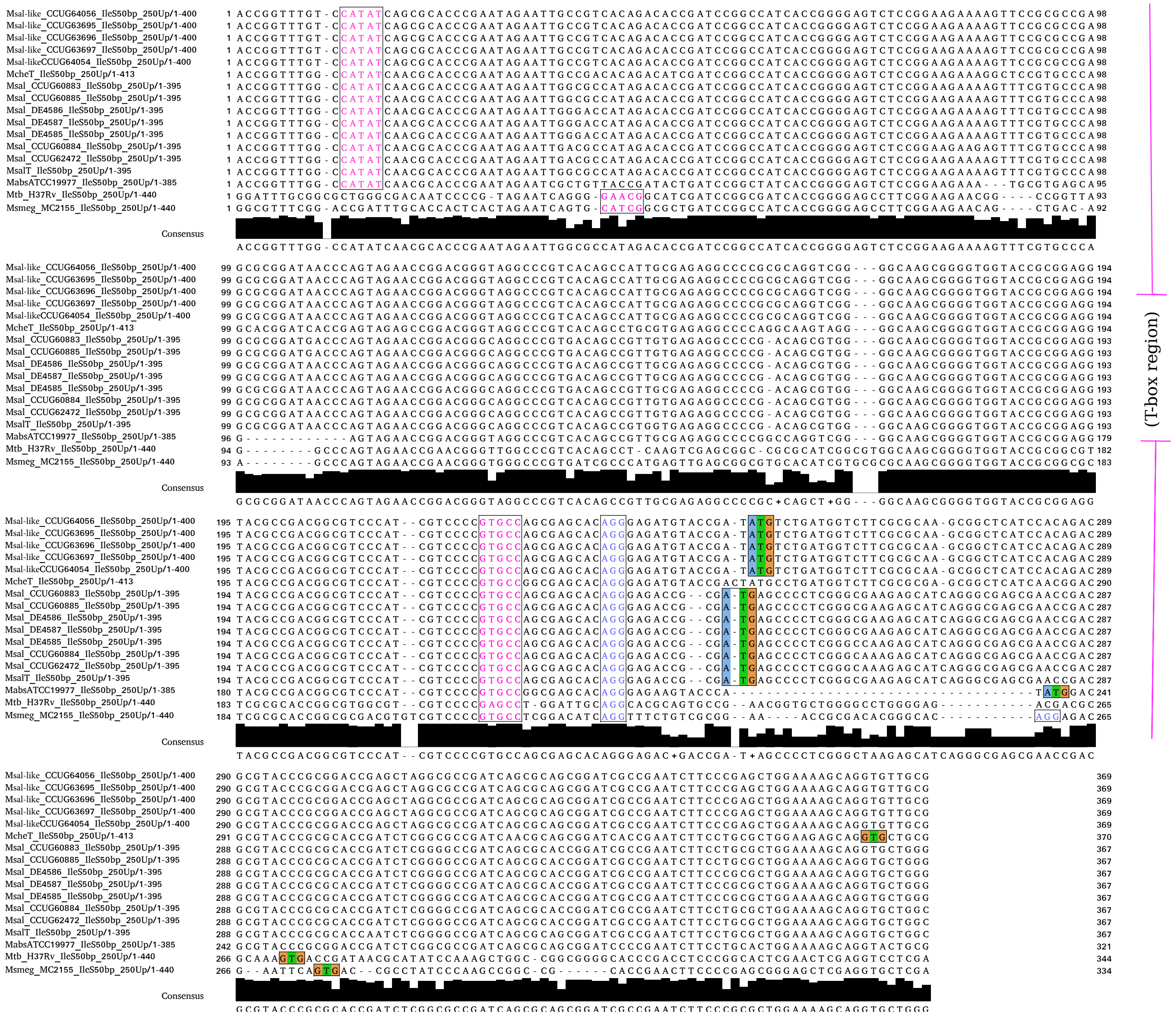

Msal-like_CCUG64056_IleS50bp_250Up/1-400
Msal-like_CCUG63695_IleS50bp_250Up/1-400
Msal-like_CCUG63696_IleS50bp_250Up/1-400
Msal-like_CCUG63697_IleS50bp_250Up/1-400
Msal-likeCCUG64054_IleS50bp_250Up/1-400
McheT_IleS50bp_250Up/1-413
Msal_CCUG60883_IleS50bp_250Up/1-395
Msal_CCUG60885_IleS50bp_250Up/1-395
Msal_DE4586_IleS50bp_250Up/1-395
Msal_DE4587_IleS50bp_250Up/1-395
Msal_DE4585_IleS50bp_250Up/1-395
Msal_CCUG60884_IleS50bp_250Up/1-395
Msal_CCUG62472_IleS50bp_250Up/1-395
MsalT_IleS50bp_250Up/1-395
MabsATCC19977_IleS50bp_250Up/1-385
Mtb_H37Rv_IleS50bp_250Up/1-440
Msmeg_MC2155_IleS50bp_250Up/1-440
Consensus
(T-box region)

Figure-S4:

c.

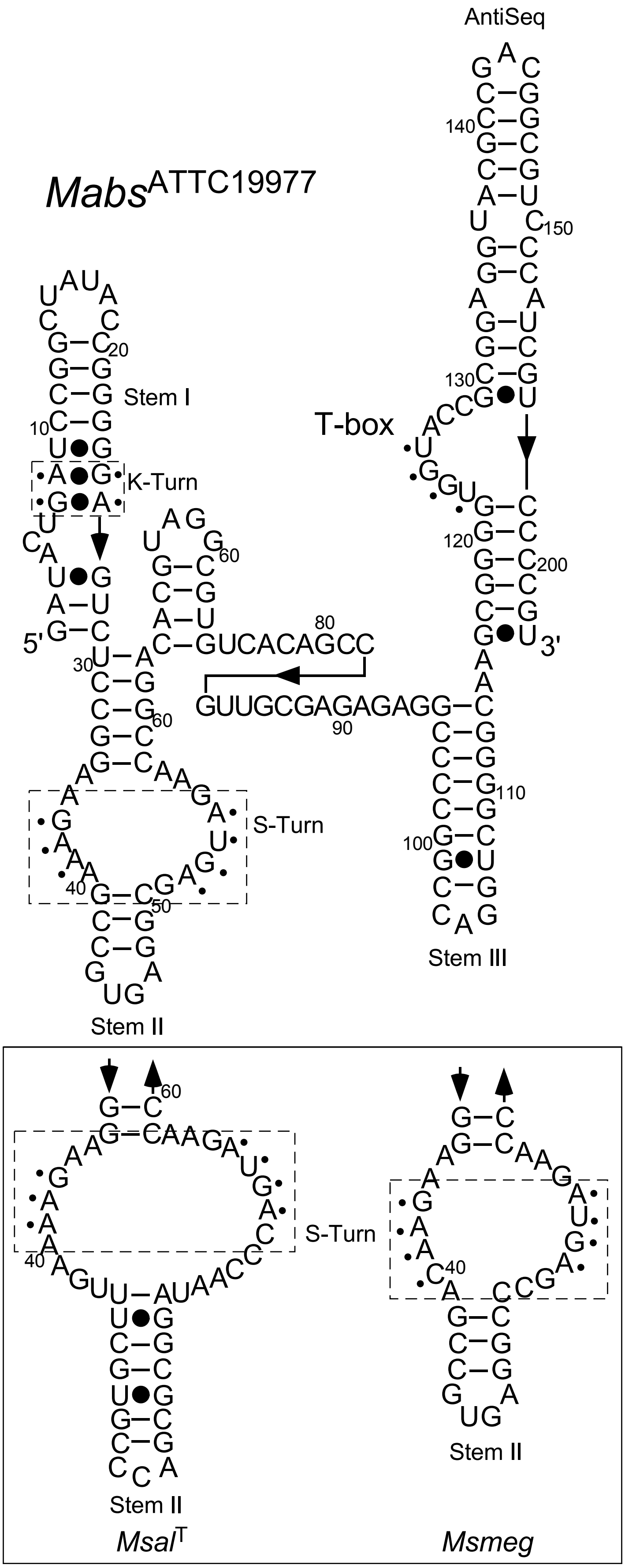

Mabs ATTC19977
AntiSeq
T-box
Stem I
K-Turn
S-Turn
Stem II
Stem III
5'
3'
S-Turn
Stem II
Stem II
Msal T
Msmeg

Figure S5 Phylogenetic relationship of MCAC-members.

(a) Phylogenetic tree based on 16S rDNA for MCAC-members as indicated.

(b) Core gene phylogenetic tree (n=623) for MCAC-members as indicated.

The phylogenetic trees were generated as described in Methods using *Mfor*^{DSM46621}, *Mulc*^{Agy99} and *Mma* M strain as outgroups.

Figure-S5:

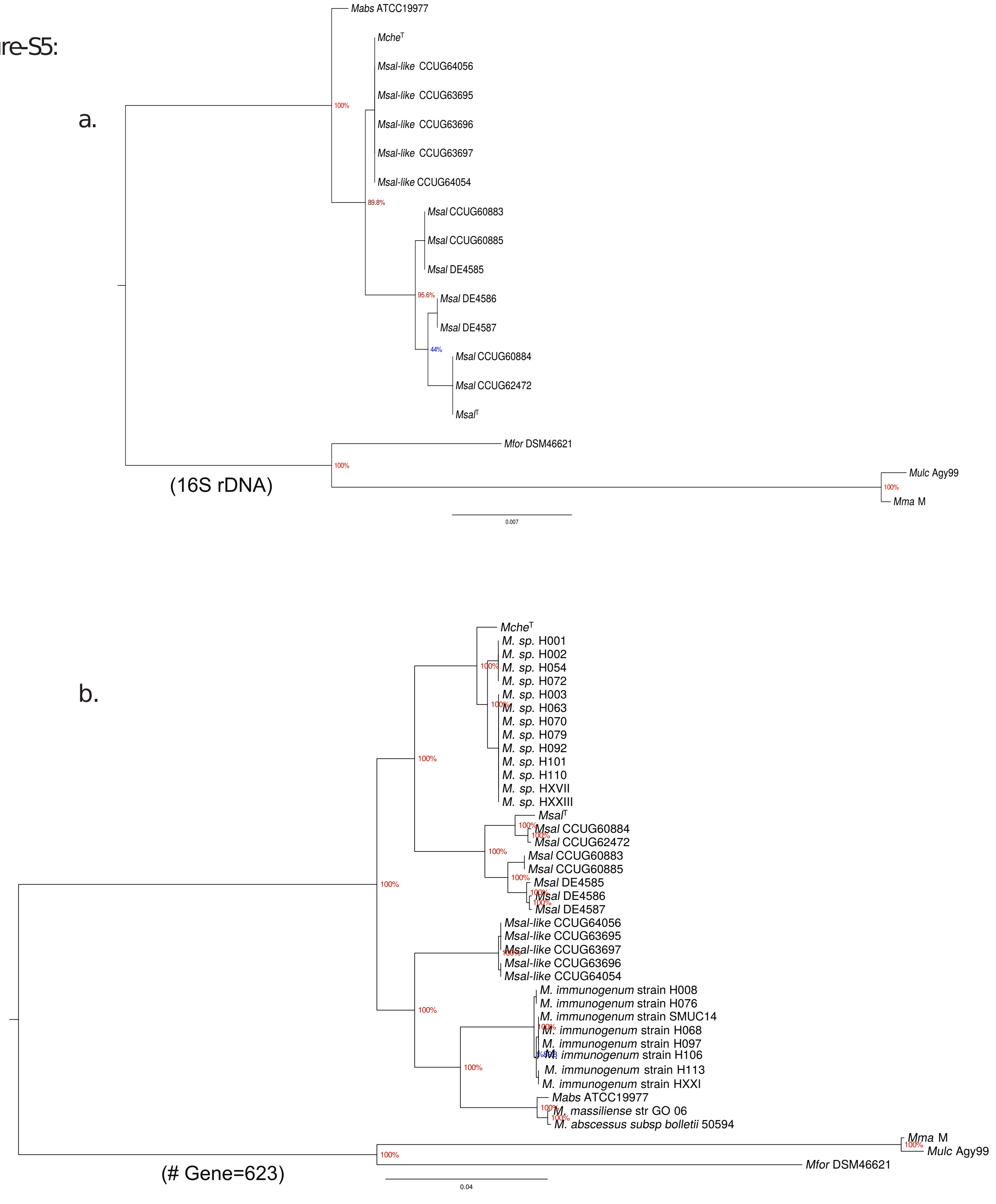

a.
Mabs ATCC19977
Mche^T
Msal-like CCUG64056
Msal-like CCUG63695
Msal-like CCUG63696
Msal-like CCUG63697
Msal-like CCUG64054
Msal CCUG60883
Msal CCUG60885
Msal DE4585
Msal DE4586
Msal DE4587
Msal CCUG60884
Msal CCUG62472
Msal^T
Mfor DSM46621
Mulc Agy99
Mma M
100%
89.8%
95.6%
44%
100%
100%
0.007
(16S rDNA)
b.
Mche^T
M. sp. H001
M. sp. H002
M. sp. H054
M. sp. H072
M. sp. H003
M. sp. H063
M. sp. H070
M. sp. H079
M. sp. H092
M. sp. H101
M. sp. H110
M. sp. HXVII
M. sp. HXXIII
Msal^T
Msal CCUG60884
Msal CCUG62472
Msal CCUG60883
Msal CCUG60885
Msal DE4585
Msal DE4586
Msal DE4587
Msal-like CCUG64056
Msal-like CCUG63695
Msal-like CCUG63697
Msal-like CCUG63696
Msal-like CCUG64054
M. immunogenum strain H008
M. immunogenum strain H076
M. immunogenum strain SMUC14
M. immunogenum strain H068
M. immunogenum strain H097
M. immunogenum strain H106
M. immunogenum strain H113
M. immunogenum strain HXXI
Mabs ATCC19977
M. massiliense str GO 06
M. abscessus subsp bolletii 50594
Mma M
Mulc Agy99
Mfor DSM46621
100%
0.04
(# Gene=623)

Figure S6 Functional classification of genes in *Mabs*^{ATCC19977}, *Mche*^{T}*, Msal*^{T} and *Msal*-like^{CCUG64054} into subsystem as indicated.

(a) Subsystem classification of genes predicted to be present in *Mabs*^{ATCC19977} (2796 genes), *Mche*^{T} (2552 genes), *Msal*^{T} (2544 genes) and *Msal*-like^{CCUG64054} (2756 genes).

(b) Subsystem classification of unique genes in *Mabs*^{ATCC19977} (299 genes), *Mche*^{T} (130 genes), *Msal*^{T} (134 genes) and *Msal*-like^{CCUG64054} (247 genes).

(c) Classification of unique genes present in *Mabs*^{ATCC19977}, *Mche*^{T}, *Msal*^{T} and *Msal*-like^{CCUG64054} in the subcategory "Amino Acids and Derivatives".

(d) Classification of unique genes present in *Mabs*^{ATCC19977}, *Mche*^{T}, *Msal*^{T} and *Msal*-like^{CCUG64054} in the subcategory "Carbohydrates".

(e) Classification of unique genes present in *Mabs*^{ATCC19977}, *Mche*^{T}, *Msal*^{T} and *Msal*-like^{CCUG64054} in the subcategory "Fatty Acids, Lipids and Isoprenoids".

For (a) – (e), one gene can be classified in more than one subsystem.

(f) Shewart control chart showing the average SNVs frequencies in *Msal* strains (n = 8). Black and red dots mark in-control SNV and out of control (hotspots) frequencies, respectively.

Figure-S6:

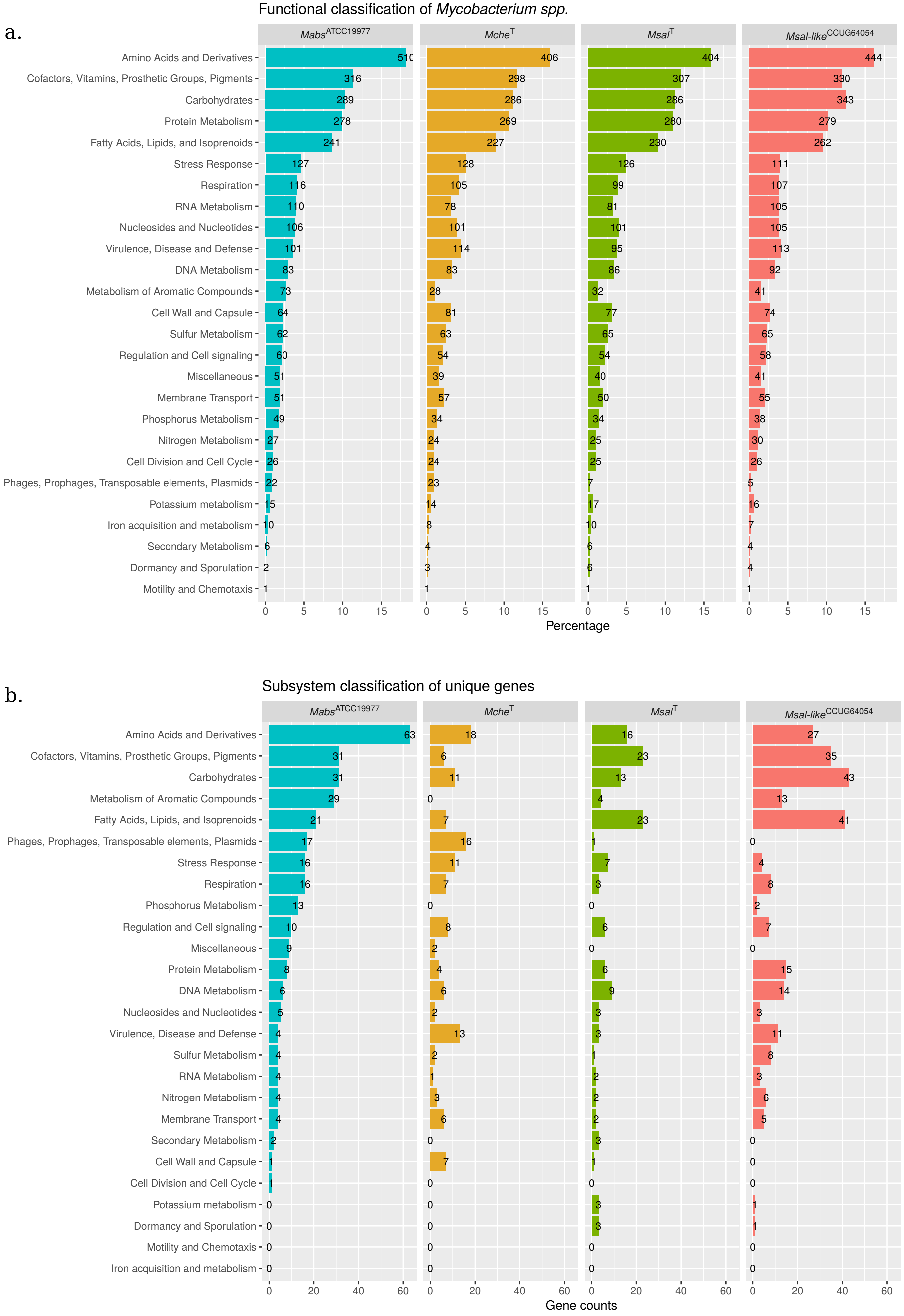

a.
Functional classification of Mycobacterium spp.
Mabs ATCC19977
Mche T
Msal T
Msal-like CCUG64054
Amino Acids and Derivatives
Cofactors, Vitamins, Prosthetic Groups, Pigments
Carbohydrates
Protein Metabolism
Fatty Acids, Lipids, and Isoprenoids
Stress Response
Respiration
RNA Metabolism
Nucleosides and Nucleotides
Virulence, Disease and Defense
DNA Metabolism
Metabolism of Aromatic Compounds
Cell Wall and Capsule
Sulfur Metabolism
Regulation and Cell signaling
Miscellaneous
Membrane Transport
Phosphorus Metabolism
Nitrogen Metabolism
Cell Division and Cell Cycle
Phages, Prophages, Transposable elements, Plasmids
Potassium metabolism
Iron acquisition and metabolism
Secondary Metabolism
Dormancy and Sporulation
Motility and Chemotaxis
Percentage
b.
Subsystem classification of unique genes
Mabs ATCC19977
Mche T
Msal T
Msal-like CCUG64054
Amino Acids and Derivatives
Cofactors, Vitamins, Prosthetic Groups, Pigments
Carbohydrates
Metabolism of Aromatic Compounds
Fatty Acids, Lipids, and Isoprenoids
Phages, Prophages, Transposable elements, Plasmids
Stress Response
Respiration
Phosphorus Metabolism
Regulation and Cell signaling
Miscellaneous
Protein Metabolism
DNA Metabolism
Nucleosides and Nucleotides
Virulence, Disease and Defense
Sulfur Metabolism
RNA Metabolism
Nitrogen Metabolism
Membrane Transport
Secondary Metabolism
Cell Wall and Capsule
Cell Division and Cell Cycle
Potassium metabolism
Dormancy and Sporulation
Motility and Chemotaxis
Iron acquisition and metabolism
Gene counts

Figure-S6:

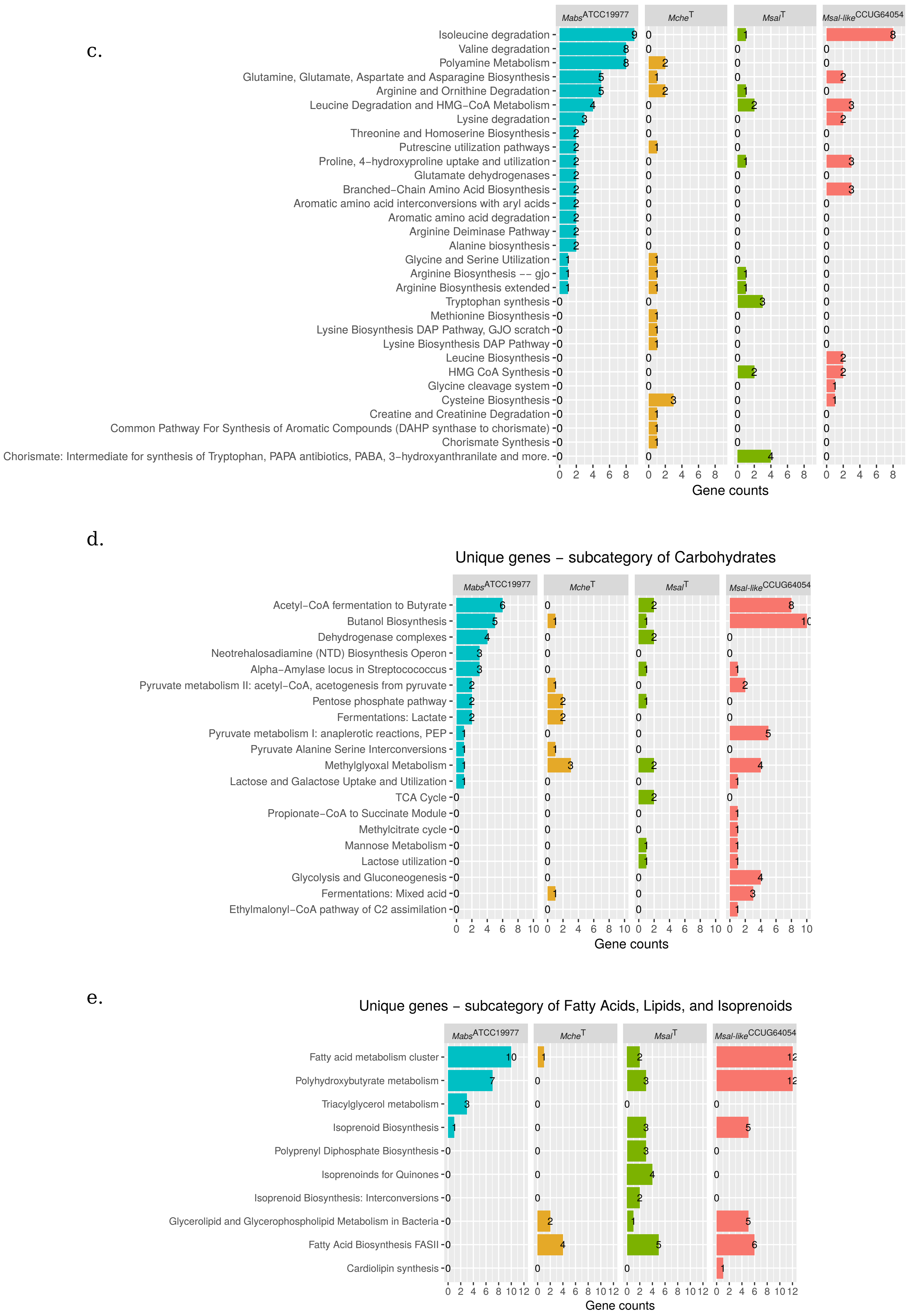
c.
Unique genes – subcategory of Amino Acids and Derivates
Mabs ATCC19977
Mche T
Msal T
Msal-like CCUG64054
Isoleucine degradation
Valine degradation
Polyamine Metabolism
Glutamine, Glutamate, Aspartate and Asparagine Biosynthesis
Arginine and Ornithine Degradation
Leucine Degradation and HMG–CoA Metabolism
Lysine degradation
Threonine and Homoserine Biosynthesis
Putrescine utilization pathways
Proline, 4–hydroxyproline uptake and utilization
Glutamate dehydrogenases
Branched–Chain Amino Acid Biosynthesis
Aromatic amino acid interconversions with aryl acids
Aromatic amino acid degradation
Arginine Deiminase Pathway
Alanine biosynthesis
Glycine and Serine Utilization
Arginine Biosynthesis –– gjo
Arginine Biosynthesis extended
Tryptophan synthesis
Methionine Biosynthesis
Lysine Biosynthesis DAP Pathway, GJO scratch
Lysine Biosynthesis DAP Pathway
Leucine Biosynthesis
HMG CoA Synthesis
Glycine cleavage system
Cysteine Biosynthesis
Creatine and Creatinine Degradation
Common Pathway For Synthesis of Aromatic Compounds (DAHP synthase to chorismate)
Chorismate Synthesis
Chorismate: Intermediate for synthesis of Tryptophan, PAPA antibiotics, PABA, 3–hydroxyanthranilate and more.
Gene counts
d.
Unique genes – subcategory of Carbohydrates
Mabs ATCC19977
Mche T
Msal T
Msal-like CCUG64054
Acetyl–CoA fermentation to Butyrate
Butanol Biosynthesis
Dehydrogenase complexes
Neotrehalosadiamine (NTD) Biosynthesis Operon
Alpha–Amylase locus in Streptocococcus
Pyruvate metabolism II: acetyl–CoA, acetogenesis from pyruvate
Pentose phosphate pathway
Fermentations: Lactate
Pyruvate metabolism I: anaplerotic reactions, PEP
Pyruvate Alanine Serine Interconversions
Methylglyoxal Metabolism
Lactose and Galactose Uptake and Utilization
TCA Cycle
Propionate–CoA to Succinate Module
Methylcitrate cycle
Mannose Metabolism
Lactose utilization
Glycolysis and Gluconeogenesis
Fermentations: Mixed acid
Ethylmalonyl–CoA pathway of C2 assimilation
Gene counts
e.
Unique genes – subcategory of Fatty Acids, Lipids, and Isoprenoids
Mabs ATCC19977
Mche T
Msal T
Msal-like CCUG64054
Fatty acid metabolism cluster
Polyhydroxybutyrate metabolism
Triacylglycerol metabolism
Isoprenoid Biosynthesis
Polyprenyl Diphosphate Biosynthesis
Isoprenoinds for Quinones
Isoprenoid Biosynthesis: Interconversions
Glycerolipid and Glycerophospholipid Metabolism in Bacteria
Fatty Acid Biosynthesis FASII
Cardiolipin synthesis
Gene counts

Figure-S6:

f.

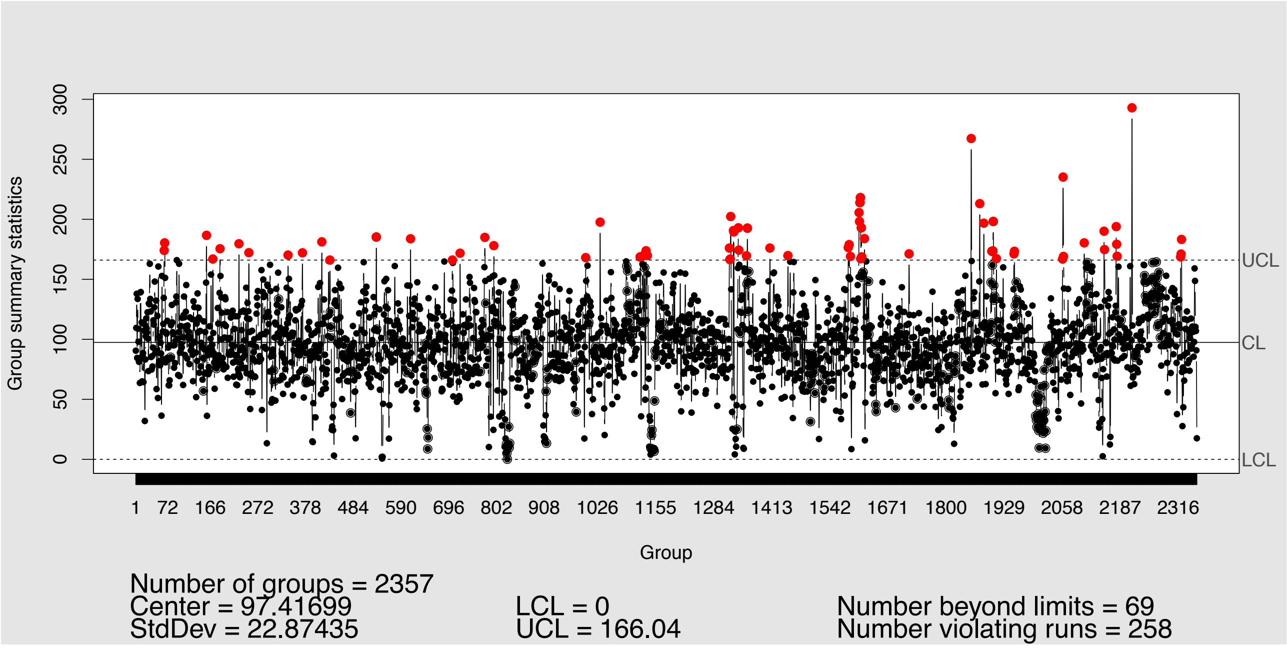
Group summary statistics
0
50
100
150
200
250
300
UCL
CL
LCL
1 72 166 272 378 484 590 696 802 908 1026 1155 1284 1413 1542 1671 1800 1929 2058 2187 2316
Group
Number of groups = 2357
Center = 97.41699
StdDev = 22.87435
LCL = 0
UCL = 166.04
Number beyond limits = 69
Number violating runs = 258

Figure S7 Horizontal gene transfer analysis in *Msal* and *Msal*-like strains, *Mche*^{T}, *Mabs*^{ATCC19977} and *Mfra*^{DSM45524T}.

(a) Bar plot showing number of predicted horizontally transferred genes. Y and X axis represent mycobacterial strains/species and predicted number of genes, respectively.

(b) Venn diagram showing common and predicted unique horizontally transferred genes in *Mabs*^{ATCC19977}, *Mche*^{T}, *Msal*^{T} and *Msal*-like^{CCUG64054}.

(c) Heat map showing the probable source of the HGT genes for *Msal* and *Msal*-like strains, *Mche*^{T}, *Mabs*^{ATCC19977} and *Mfra*^{DSM45524T}. The vertical tree represents the heat map clustering of the column wise dendogram. Color code, see top left corner of the plot.

Figure-S7

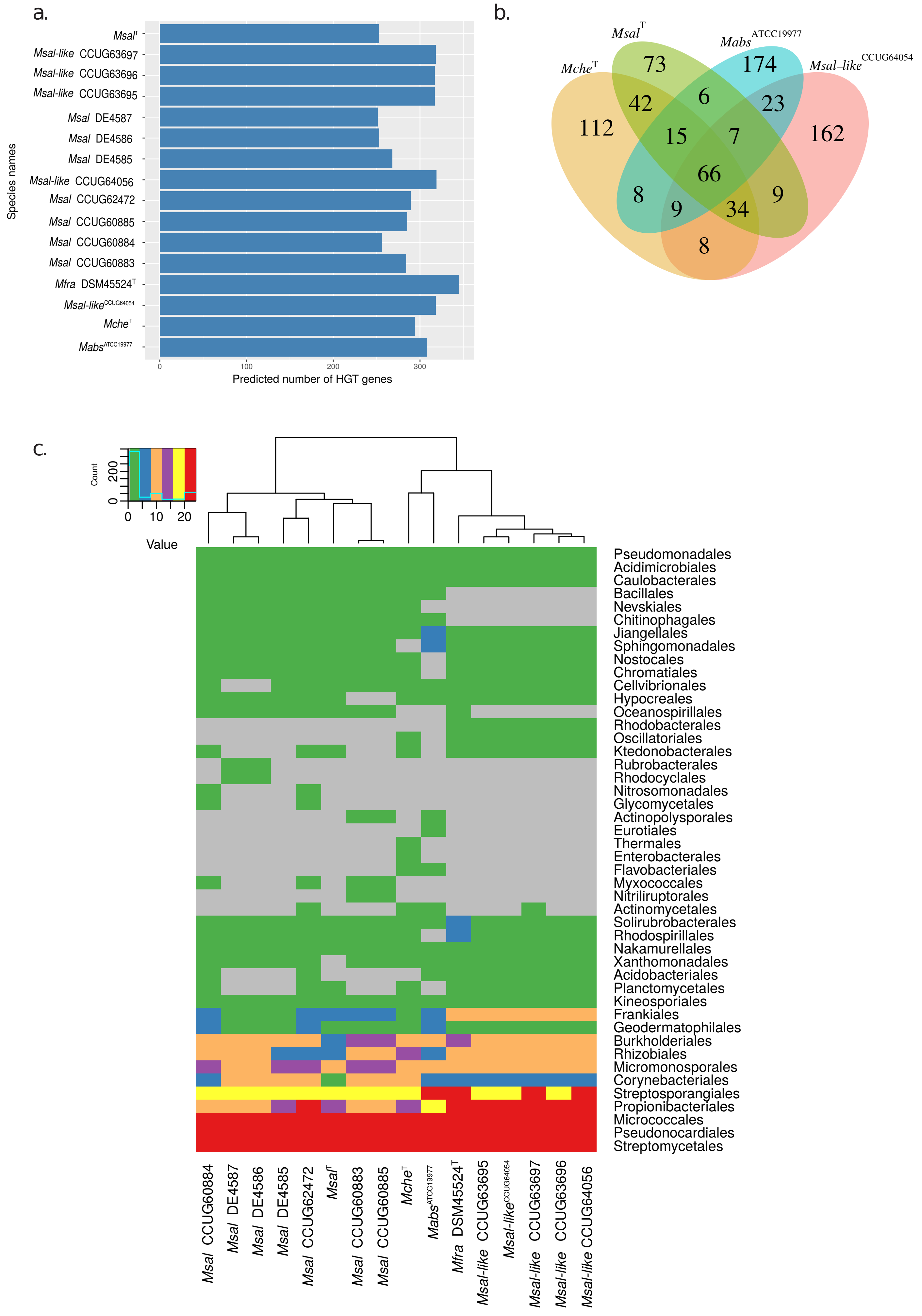

a.
Species names
MsalT
Msal-like CCUG63697
Msal-like CCUG63696
Msal-like CCUG63695
Msal DE4587
Msal DE4586
Msal DE4585
Msal-like CCUG64056
Msal CCUG62472
Msal CCUG60885
Msal CCUG60884
Msal CCUG60883
Mfra DSM45524T
Msal-likeCCUG64054
McheT
MabsATCC19977
0
100
200
300
Predicted number of HGT genes
b.
MsalT
MabsATCC19977
McheT
Msal–likeCCUG64054
73
174
112
42
6
23
162
15
7
66
8
9
34
9
8
c.
Count
0
200
0
10
20
Value
Pseudomonadales
Acidimicrobiales
Caulobacterales
Bacillales
Nevskiales
Chitinophagales
Jiangellales
Sphingomonadales
Nostocales
Chromatiales
Cellvibrionales
Hypocreales
Oceanospirillales
Rhodobacterales
Oscillatoriales
Ktedonobacterales
Rubrobacterales
Rhodocyclales
Nitrosomonadales
Glycomycetales
Actinopolysporales
Eurotiales
Thermales
Enterobacterales
Flavobacteriales
Myxococcales
Nitriliruptorales
Actinomycetales
Solirubrobacterales
Rhodospirillales
Nakamurellales
Xanthomonadales
Acidobacteriales
Planctomycetales
Kineosporiales
Frankiales
Geodermatophilales
Burkholderiales
Rhizobiales
Micromonosporales
Corynebacteriales
Streptosporangiales
Propionibacteriales
Micrococcales
Pseudonocardiales
Streptomycetales
Msal CCUG60884
Msal DE4587
Msal DE4586
Msal DE4585
Msal CCUG62472
MsalT
Msal CCUG60883
Msal CCUG60885
McheT
MabsATCC19977
Mfra DSM45524T
Msal-like CCUG63695
Msal-likeCCUG64054
Msal-like CCUG63697
Msal-like CCUG63696
Msal-like CCUG64056

Figure S8 Heat map showing distribution of virulence genes in *Mabs*$^{\text{ATCC19977}}$, *Mche*^{T}, *Msal*^{T} and *Msal*-like$^{\text{CCUG64054}}$ and other mycobacteria as indicated. The vertical tree represents the heat map clustering of the column wise dendogram. Green = present and gray = absent.

Figure-S8:

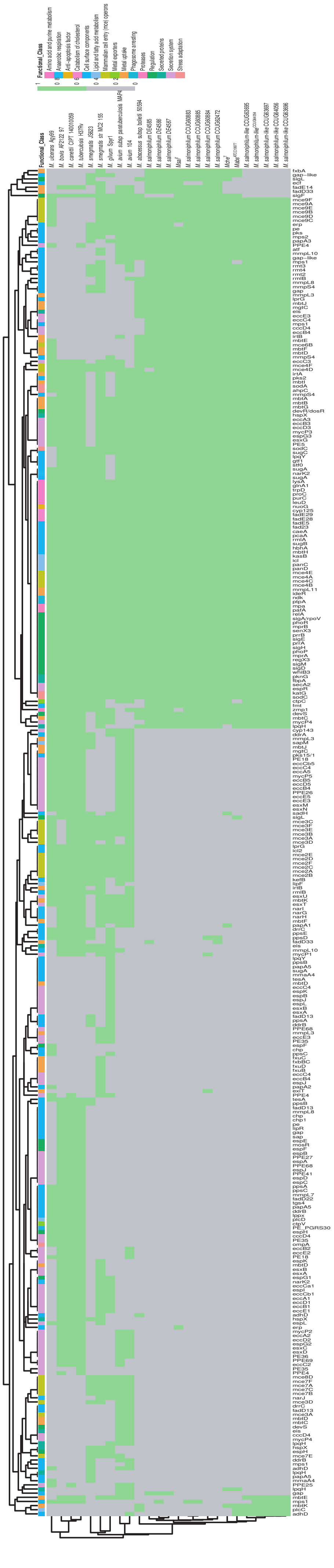

Figure S9 Compilation of tRNA genes predicted in MCAC-members.

(a) Heat map showing presence (green) and absence (light grey) of tRNA genes in MCAC-members indicated below. The clustered tRNA gene names with mycobacteria strain/species and tRNA isoacceptor name, *e.g.*, *Msal*-DSM43276_7tRNA_Ser_CGA, are listed on the right. The horizontal and vertical trees represent the heat map clustering of the column and row wise dendograms.

(b) *Mche*^{T} *vs Msal*^{T}. Blue and red marked tRNA genes refers to transcription from the positive and negative strands, respectively. Blue lines mark that the locations of the tRNA genes have not shifted.

(c) *Mabs*^{ATCC19977} *vs Msal*-like^{CCUG64054}. Blue and red marked tRNA genes, and blue lines as in (b; see above), while red lines mark tRNA genes that have shifted position on the chromosome. Of note, the *Msal*-like^{CCUG64054} is a draft genome while *Mabs*^{ATCC19977} is a complete genome.

(d) *Mabs*^{ATCC19977} *vs Mche*^{T}. Blue and red marked tRNA genes, and blue lines as in (b; see above).

(e) Analysis of the gene synteny for a tRNA gene cluster encompassing nine genes in *Msal* and *Msal*-like strains, and *Mche*^{T} and *Mabs*^{ATCC19977} as indicated. The tRNA genes are marked in red and the vertical boxes marked in brown highlight homologous genes. For further details see main text and Figs 6c, S1a and S9a.

(f) Sequence alignments of the common and "extra" tRNA genes as indicated. With respect to tRNA^{Leu}CAG the arrows mark residues forming the amino acid acceptor-stem, D-stem, anticodon-stem and T-stem. For details see the main text.

Figure-S9

a)

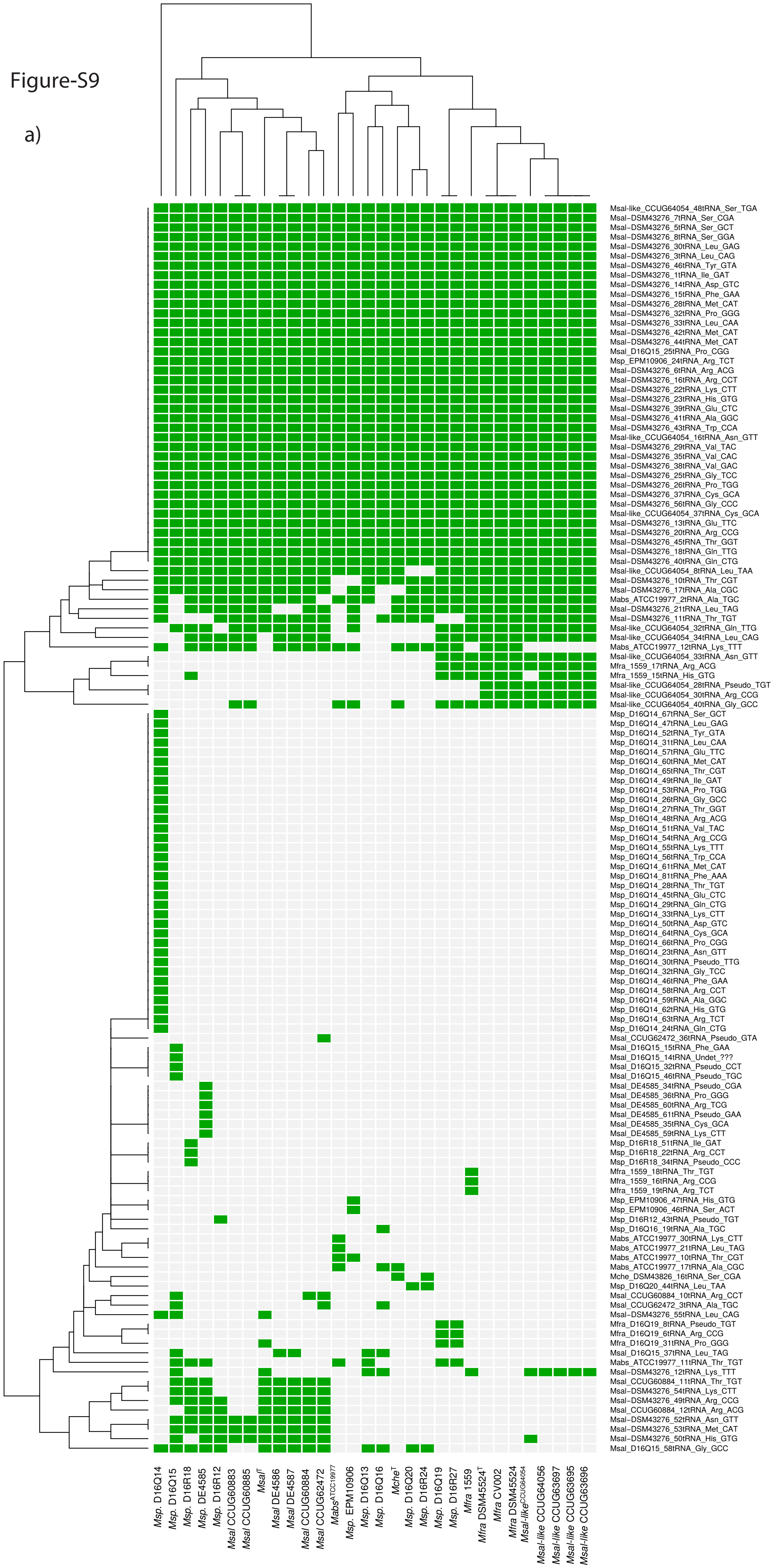
Msal-like_CCUG64054_48tRNA_Ser_TGA
Msal-DSM43276_7tRNA_Ser_CGA
Msal-DSM43276_5tRNA_Ser_GCT
Msal-DSM43276_8tRNA_Ser_GGA
Msal-DSM43276_30tRNA_Leu_GAG
Msal-DSM43276_3tRNA_Leu_CAG
Msal-DSM43276_46tRNA_Tyr_GTA
Msal-DSM43276_1tRNA_Ile_GAT
Msal-DSM43276_14tRNA_Asp_GTC
Msal-DSM43276_15tRNA_Phe_GAA
Msal-DSM43276_28tRNA_Met_CAT
Msal-DSM43276_32tRNA_Pro_GGG
Msal-DSM43276_33tRNA_Leu_CAA
Msal-DSM43276_42tRNA_Met_CAT
Msal-DSM43276_44tRNA_Met_CAT
Msal_D16Q15_25tRNA_Pro_CGG
Msp_EPM10906_24tRNA_Arg_TCT
Msal-DSM43276_6tRNA_Arg_ACG
Msal-DSM43276_16tRNA_Arg_CCT
Msal-DSM43276_22tRNA_Lys_CTT
Msal-DSM43276_23tRNA_His_GTG
Msal-DSM43276_39tRNA_Glu_CTC
Msal-DSM43276_41tRNA_Ala_GGC
Msal-DSM43276_43tRNA_Trp_CCA
Msal-like_CCUG64054_16tRNA_Asn_GTT
Msal-DSM43276_29tRNA_Val_TAC
Msal-DSM43276_35tRNA_Val_CAC
Msal-DSM43276_38tRNA_Val_GAC
Msal-DSM43276_25tRNA_Gly_TCC
Msal-DSM43276_26tRNA_Pro_TGG
Msal-DSM43276_37tRNA_Cys_GCA
Msal-DSM43276_56tRNA_Gly_CCC
Msal-like_CCUG64054_37tRNA_Cys_GCA
Msal-DSM43276_13tRNA_Glu_TTC
Msal-DSM43276_20tRNA_Arg_CCG
Msal-DSM43276_45tRNA_Thr_GGT
Msal-DSM43276_18tRNA_Gln_TTG
Msal-DSM43276_40tRNA_Gln_CTG
Msal-like_CCUG64054_8tRNA_Leu_TAA
Msal-DSM43276_10tRNA_Thr_CGT
Msal-DSM43276_17tRNA_Ala_CGC
Mabs_ATCC19977_2tRNA_Ala_TGC
Msal-DSM43276_21tRNA_Leu_TAG
Msal-DSM43276_11tRNA_Thr_TGT
Msal-like_CCUG64054_32tRNA_Gln_TTG
Msal-like_CCUG64054_34tRNA_Leu_CAG
Mabs_ATCC19977_12tRNA_Lys_TTT
Msal-like_CCUG64054_33tRNA_Asn_GTT
Mfra_1559_17tRNA_Arg_ACG
Mfra_1559_15tRNA_His_GTG
Msal-like_CCUG64054_28tRNA_Pseudo_TGT
Msal-like_CCUG64054_30tRNA_Arg_CCG
Msal-like_CCUG64054_40tRNA_Gly_GCC
Msp_D16Q14_67tRNA_Ser_GCT
Msp_D16Q14_47tRNA_Leu_GAG
Msp_D16Q14_52tRNA_Tyr_GTA
Msp_D16Q14_31tRNA_Leu_CAA
Msp_D16Q14_57tRNA_Glu_TTC
Msp_D16Q14_60tRNA_Met_CAT
Msp_D16Q14_65tRNA_Thr_CGT
Msp_D16Q14_49tRNA_Ile_GAT
Msp_D16Q14_53tRNA_Pro_TGG
Msp_D16Q14_26tRNA_Gly_GCC
Msp_D16Q14_27tRNA_Thr_GGT
Msp_D16Q14_48tRNA_Arg_ACG
Msp_D16Q14_51tRNA_Val_TAC
Msp_D16Q14_54tRNA_Arg_CCG
Msp_D16Q14_55tRNA_Lys_TTT
Msp_D16Q14_56tRNA_Trp_CCA
Msp_D16Q14_61tRNA_Met_CAT
Msp_D16Q14_81tRNA_Phe_AAA
Msp_D16Q14_28tRNA_Thr_TGT
Msp_D16Q14_45tRNA_Glu_CTC
Msp_D16Q14_29tRNA_Gln_CTG
Msp_D16Q14_33tRNA_Lys_CTT
Msp_D16Q14_50tRNA_Asp_GTC
Msp_D16Q14_64tRNA_Cys_GCA
Msp_D16Q14_66tRNA_Pro_CGG
Msp_D16Q14_23tRNA_Asn_GTT
Msp_D16Q14_30tRNA_Pseudo_TTG
Msp_D16Q14_32tRNA_Gly_TCC
Msp_D16Q14_46tRNA_Phe_GAA
Msp_D16Q14_58tRNA_Arg_CCT
Msp_D16Q14_59tRNA_Ala_GGC
Msp_D16Q14_62tRNA_His_GTG
Msp_D16Q14_63tRNA_Arg_TCT
Msp_D16Q14_24tRNA_Gln_CTG
Msal_CCUG62472_36tRNA_Pseudo_GTA
Msal_D16Q15_15tRNA_Phe_GAA
Msal_D16Q15_14tRNA_Undet_???
Msal_D16Q15_32tRNA_Pseudo_CCT
Msal_D16Q15_46tRNA_Pseudo_TGC
Msal_DE4585_34tRNA_Pseudo_CGA
Msal_DE4585_36tRNA_Pro_GGG
Msal_DE4585_60tRNA_Arg_TCG
Msal_DE4585_61tRNA_Pseudo_GAA
Msal_DE4585_35tRNA_Cys_GCA
Msal_DE4585_59tRNA_Lys_CTT
Msp_D16R18_51tRNA_Ile_GAT
Msp_D16R18_22tRNA_Arg_CCT
Msp_D16R18_34tRNA_Pseudo_CCC
Mfra_1559_18tRNA_Thr_TGT
Mfra_1559_16tRNA_Arg_CCG
Mfra_1559_19tRNA_Arg_TCT
Msp_EPM10906_47tRNA_His_GTG
Msp_EPM10906_46tRNA_Ser_ACT
Msp_D16R12_43tRNA_Pseudo_TGT
Msp_D16Q16_19tRNA_Ala_TGC
Mabs_ATCC19977_30tRNA_Lys_CTT
Mabs_ATCC19977_21tRNA_Leu_TAG
Mabs_ATCC19977_10tRNA_Thr_CGT
Mabs_ATCC19977_17tRNA_Ala_CGC
Mche_DSM43826_16tRNA_Ser_CGA
Msp_D16Q20_44tRNA_Leu_TAA
Msal_CCUG60884_10tRNA_Arg_CCT
Msal_CCUG62472_3tRNA_Ala_TGC
Msal-DSM43276_55tRNA_Leu_CAG
Mfra_D16Q19_8tRNA_Pseudo_TGT
Mfra_D16Q19_6tRNA_Arg_CCG
Mfra_D16Q19_31tRNA_Pro_GGG
Msal_D16Q15_37tRNA_Leu_TAG
Mabs_ATCC19977_11tRNA_Thr_TGT
Msal-DSM43276_12tRNA_Lys_TTT
Msal_CCUG60884_11tRNA_Thr_TGT
Msal-DSM43276_54tRNA_Lys_CTT
Msal-DSM43276_49tRNA_Arg_CCG
Msal_CCUG60884_12tRNA_Arg_ACG
Msal-DSM43276_52tRNA_Asn_GTT
Msal-DSM43276_53tRNA_Met_CAT
Msal-DSM43276_50tRNA_His_GTG
Msal_D16Q15_58tRNA_Gly_GCC
Msp. D16Q14
Msp. D16Q15
Msp. D16R18
Msp. DE4585
Msp. D16R12
Msal CCUG60883
Msal CCUG60885
Msal T
Msal DE4586
Msal DE4587
Msal CCUG60884
Msal CCUG62472
Mabs ATCC19977
Msp. EPM10906
Msp. D16Q13
Msp. D16Q16
Mche T
Msp. D16Q20
Msp. D16R24
Msp. D16Q19
Msp. D16R27
Mfra 1559
Mfra DSM45524 T
Mfra CV002
Mfra DSM45524
Msal-like CCUG64054
Msal-like CCUG64056
Msal-like CCUG63697
Msal-like CCUG63695
Msal-like CCUG63696

Figure-S9

b)

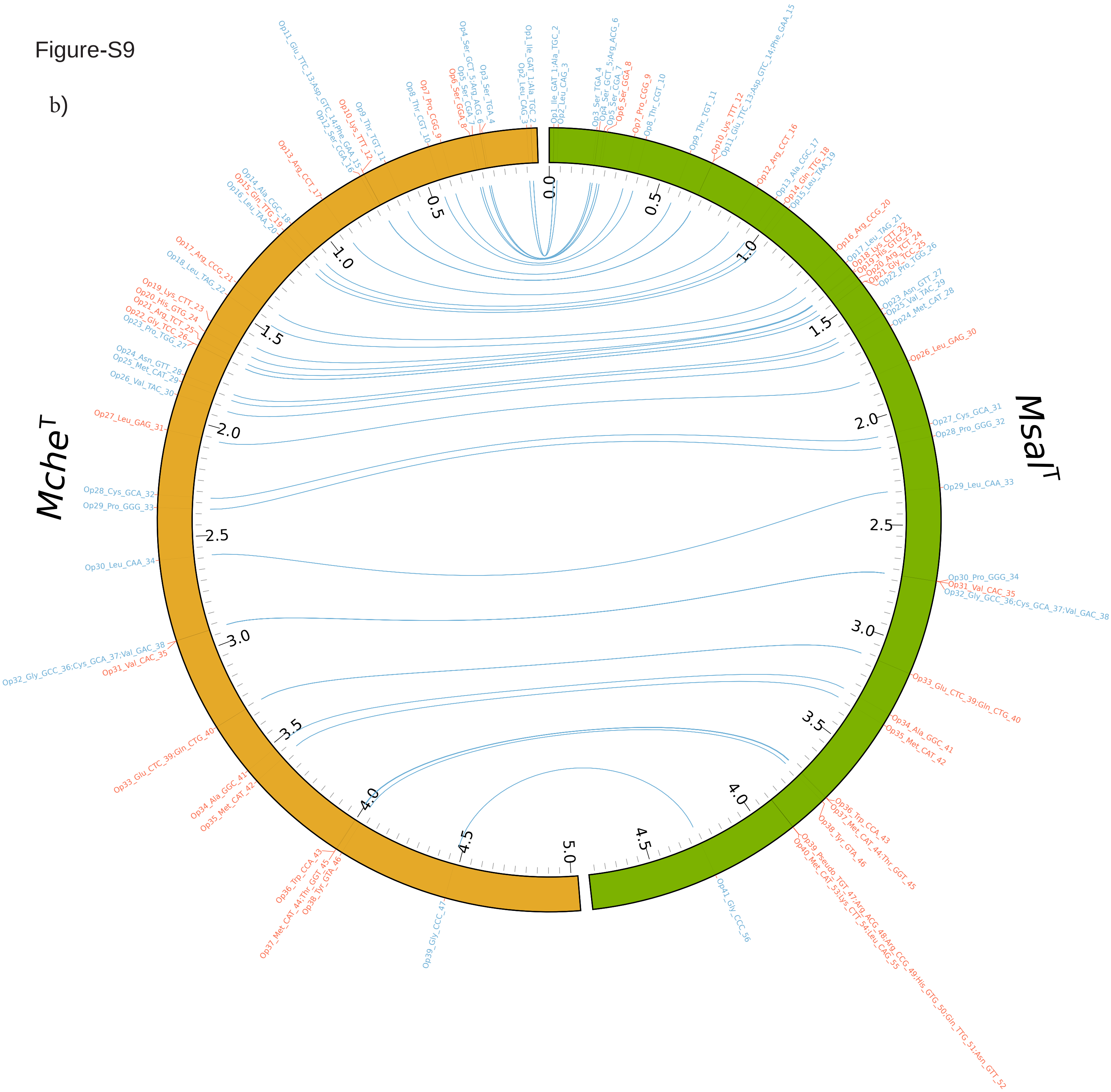

Figure-S9

c)

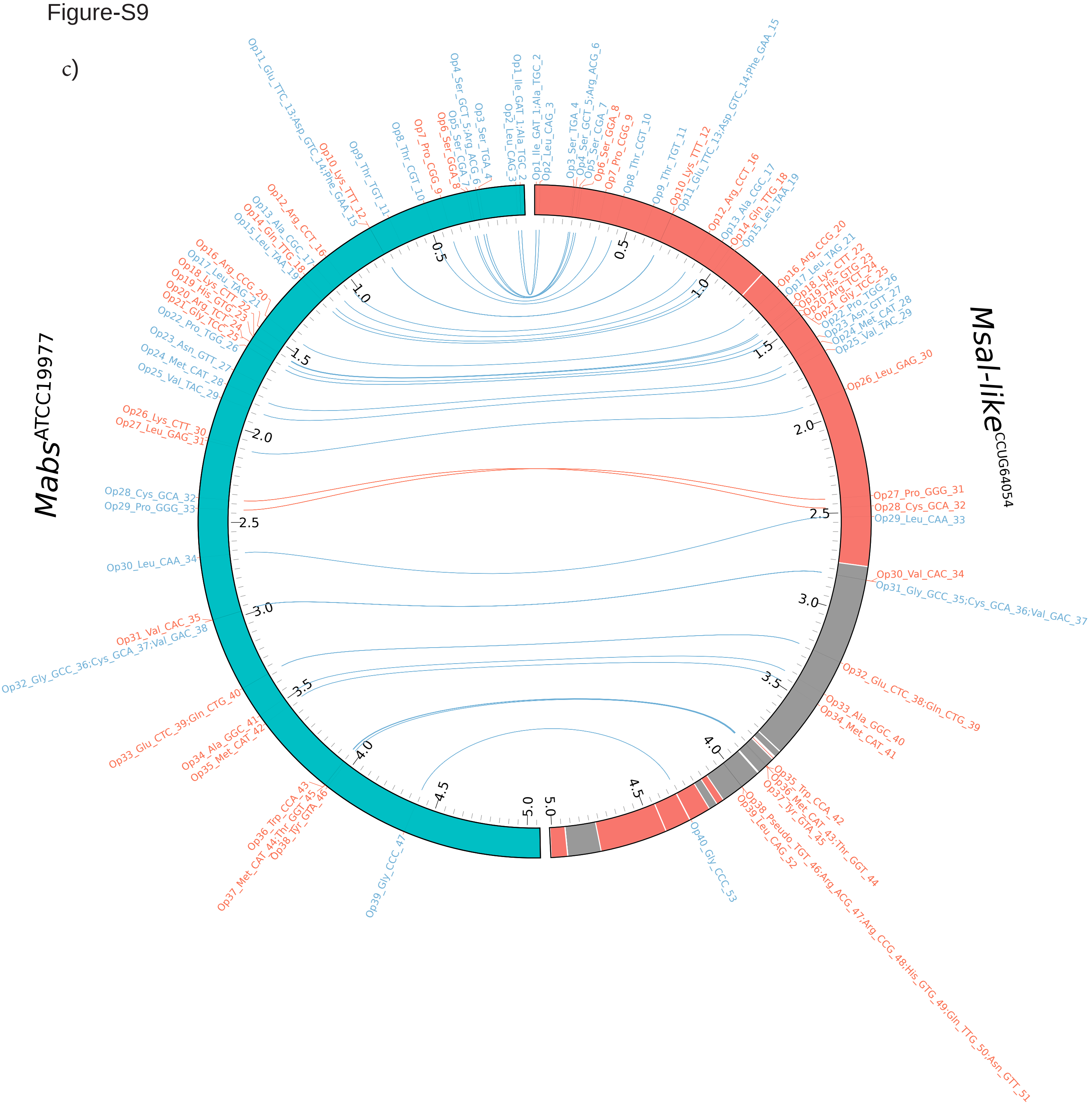

Mabs ATCC19977
Msal-like CCUG64054
Op1_Ile_GAT_1;Ala_TGC_2
Op2_Leu_CAG_3
Op3_Ser_TGA_4
Op4_Ser_GCT_5;Arg_ACG_6
Op5_Ser_CGA_7
Op6_Ser_GGA_8
Op7_Pro_CGG_9
Op8_Thr_CGT_10
Op9_Thr_TGT_11
Op10_Lys_TTT_12
Op11_Glu_TTC_13;Asp_GTC_14;Phe_GAA_15
Op12_Arg_CCT_16
Op13_Ala_CGC_17
Op14_Gln_TTG_18
Op15_Leu_TAA_19
Op16_Arg_CCG_20
Op17_Leu_TAG_21
Op18_Lys_CTT_22
Op19_His_GTG_23
Op20_Arg_TCT_24
Op21_Gly_TCC_25
Op22_Pro_TGG_26
Op23_Asn_GTT_27
Op24_Met_CAT_28
Op25_Val_TAC_29
Op26_Lys_CTT_30
Op27_Leu_GAG_31
Op28_Cys_GCA_32
Op29_Pro_GGG_33
Op30_Leu_CAA_34
Op31_Val_CAC_35
Op32_Gly_GCC_36;Cys_GCA_37;Val_GAC_38
Op33_Glu_CTC_39;Gln_CTG_40
Op34_Ala_GGC_41
Op35_Met_CAT_42
Op36_Trp_CCA_43
Op37_Met_CAT_44;Thr_GGT_45
Op38_Tyr_GTA_46
Op39_Gly_CCC_47
Op26_Leu_GAG_30
Op27_Pro_GGG_31
Op28_Cys_GCA_32
Op29_Leu_CAA_33
Op30_Val_CAC_34
Op31_Gly_GCC_35;Cys_GCA_36;Val_GAC_37
Op32_Glu_CTC_38;Gln_CTG_39
Op33_Ala_GGC_40
Op34_Met_CAT_41
Op35_Trp_CCA_42
Op36_Met_CAT_43;Thr_GGT_44
Op37_Tyr_GTA_45
Op38_Pseudo_TGT_46;Arg_ACG_47;Arg_CCG_48;His_GTG_49;Gln_TTG_50;Asn_GTT_51
Op39_Leu_CAG_52
Op40_Gly_CCC_53
0.5
1.0
1.5
2.0
2.5
3.0
3.5
4.0
4.5
5.0

Figure-S9

d)

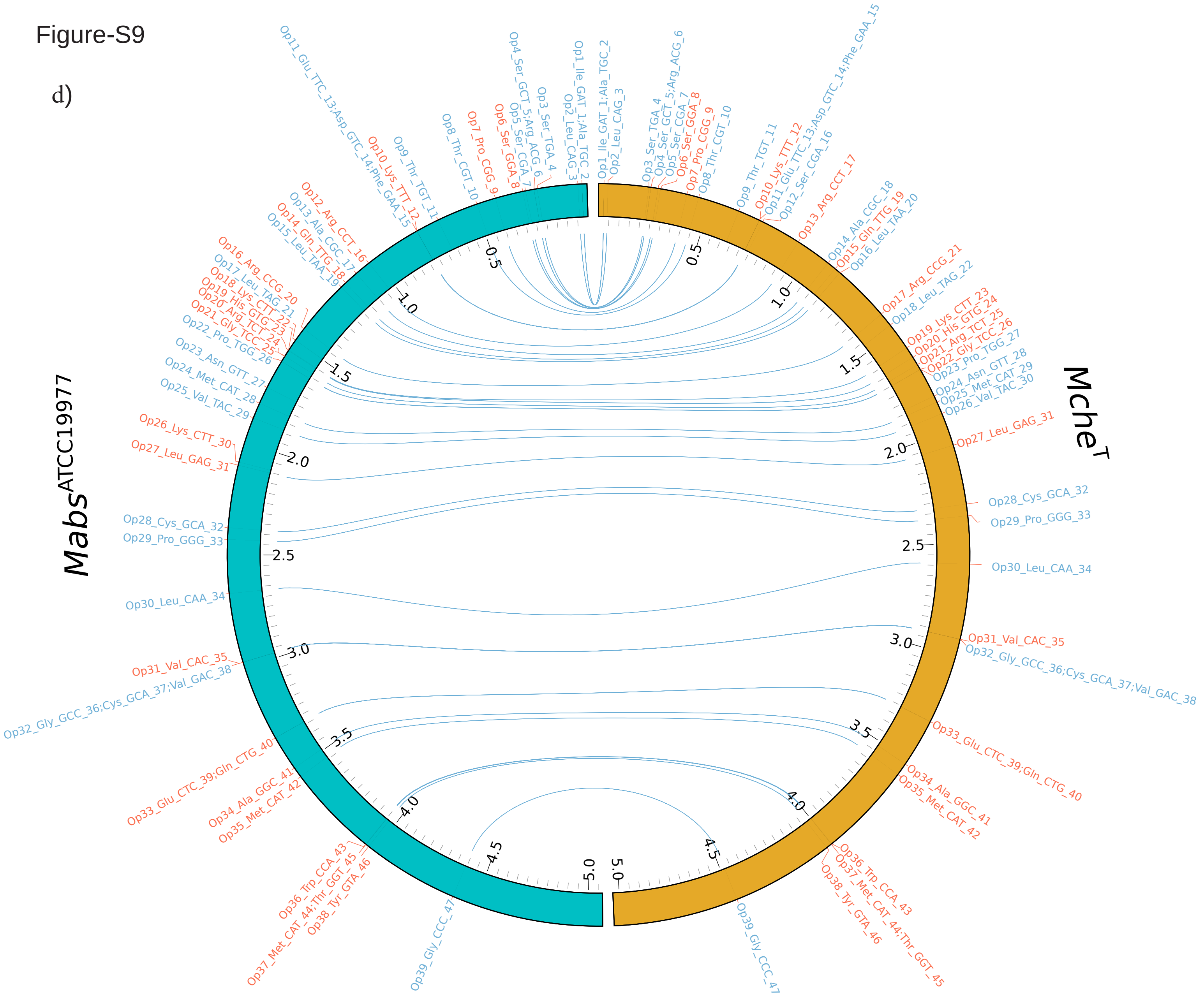

Figure-S9

e)

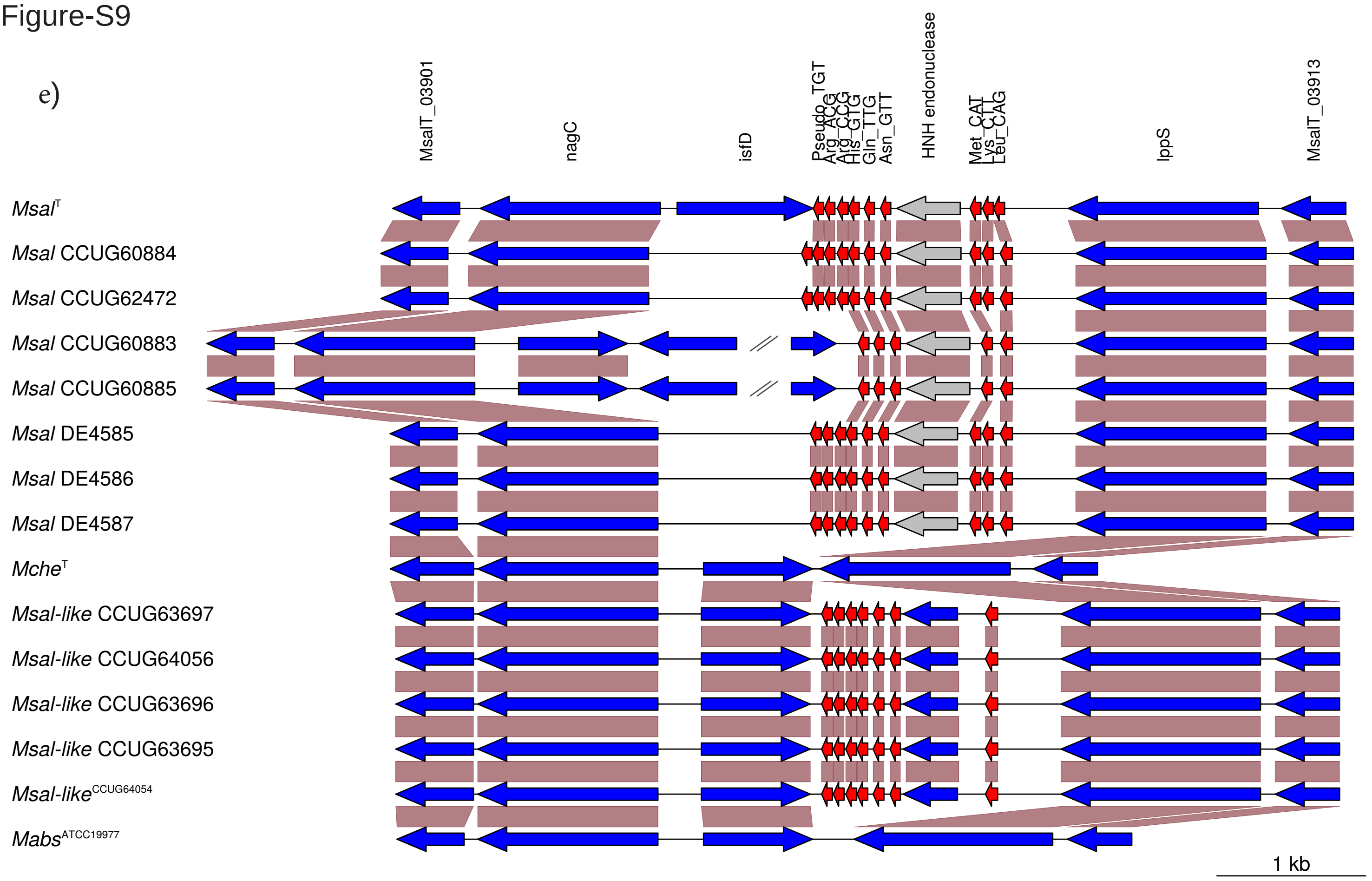
MsalT_03901
nagC
isfD
Pseudo_TGT
Arg_ACG
Arg_CCG
His_GTG
Gln_TTG
Asn_GTT
HNH endonuclease
Met_CAT
Lys_CTT
Leu_CAG
lppS
MsalT_03913
Msal[T]
Msal CCUG60884
Msal CCUG62472
Msal CCUG60883
Msal CCUG60885
Msal DE4585
Msal DE4586
Msal DE4587
Mche[T]
Msal-like CCUG63697
Msal-like CCUG64056
Msal-like CCUG63696
Msal-like CCUG63695
Msal-like[CCUG64054]
Mabs[ATCC19977]
1 kb

Figure-S9 f)

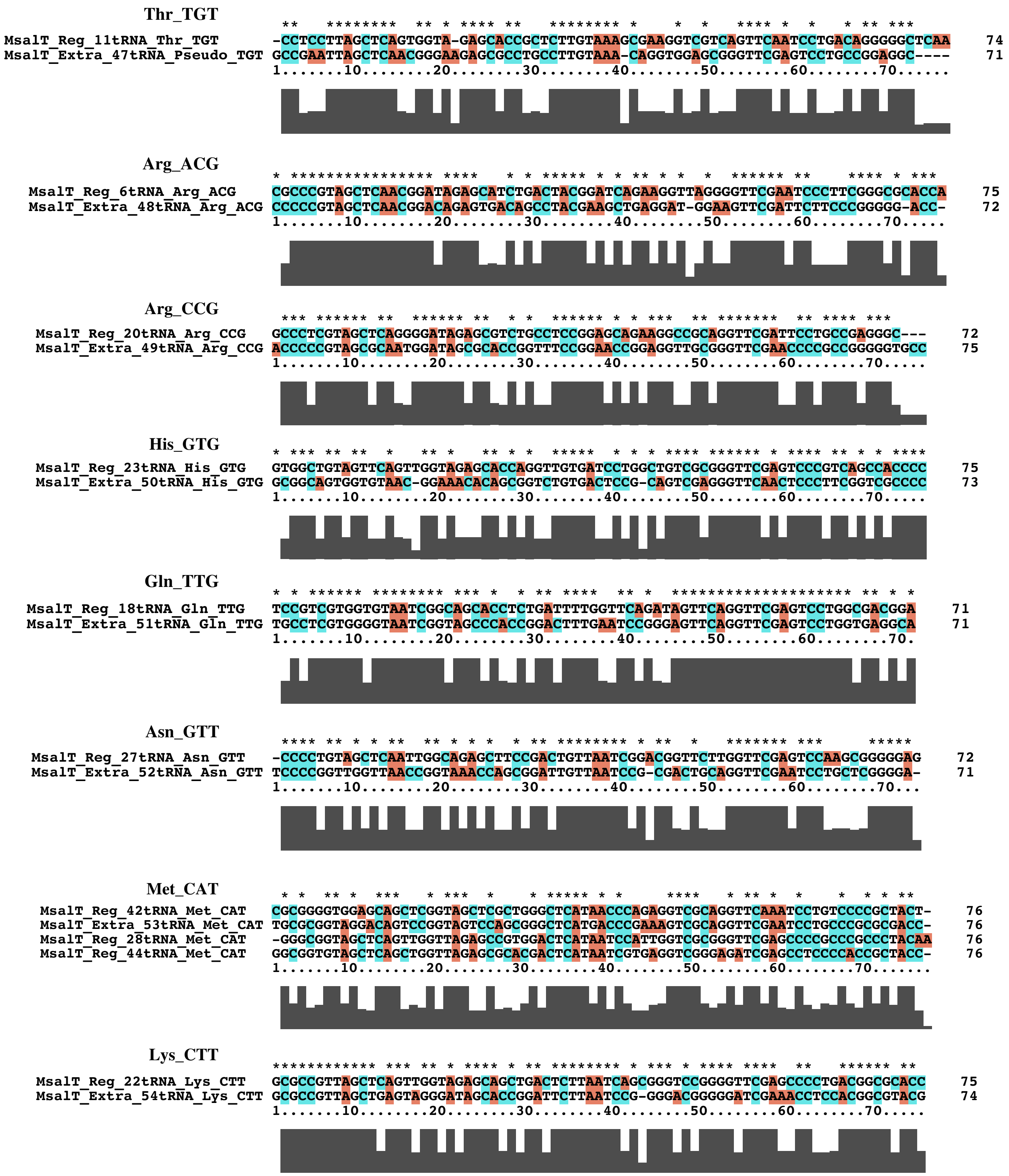

Leu_CAG

MsalT_Reg_3tRNA_Leu_CAG

```
GGCGAGTGGCGGAATGGCaGACGCGCTGGCTTCAGGTGCCAGTGTCCTTCGGGACGTGGGGGTTCAAGTCCCCCTTCGCccaCA
>>>>>>..>>>.........<<<.>>>>>......<<<<<.>>>>....<<<<..>>>>>......<<<<<<<<<<...<.
```

MsalT_Extra_55tRNA_Leu_CAG

```
GCCCCTCTGGCCCAACTGGAaGAGGCGTCCCGTTCAGGGCGGGAAGGtTCCTGGTTCGAATCCAGGGAGGGGTA
>>>>>>>..>>>...........<<<.>>>>>......<<<<<....>>>>>.......<<<<<<<<<<<<.
```

Figure S10 Extended ANI analysis including *Mycobacterium saopaulense*.

(a) Heat map showing ANI values for "all-versus-all" *Msal* and *Msal*-like strains, *Mabs*$^{\text{ATCC19977}}$, *Mche*^{T}, *M. saopaulense*, *Mma* M strain, *Mulc*$^{\text{Agy99}}$ and *Mfor*$^{\text{DSM46621}}$ as indicated. ANI values were clustered based on unsupervised hierarchical clustering (see Methods, main text and Fig 2).

(b) Dendogram, extracted from the heat map shown in (a), displaying clustering of different strains / based on ANI values.

Figure-S10:

a.

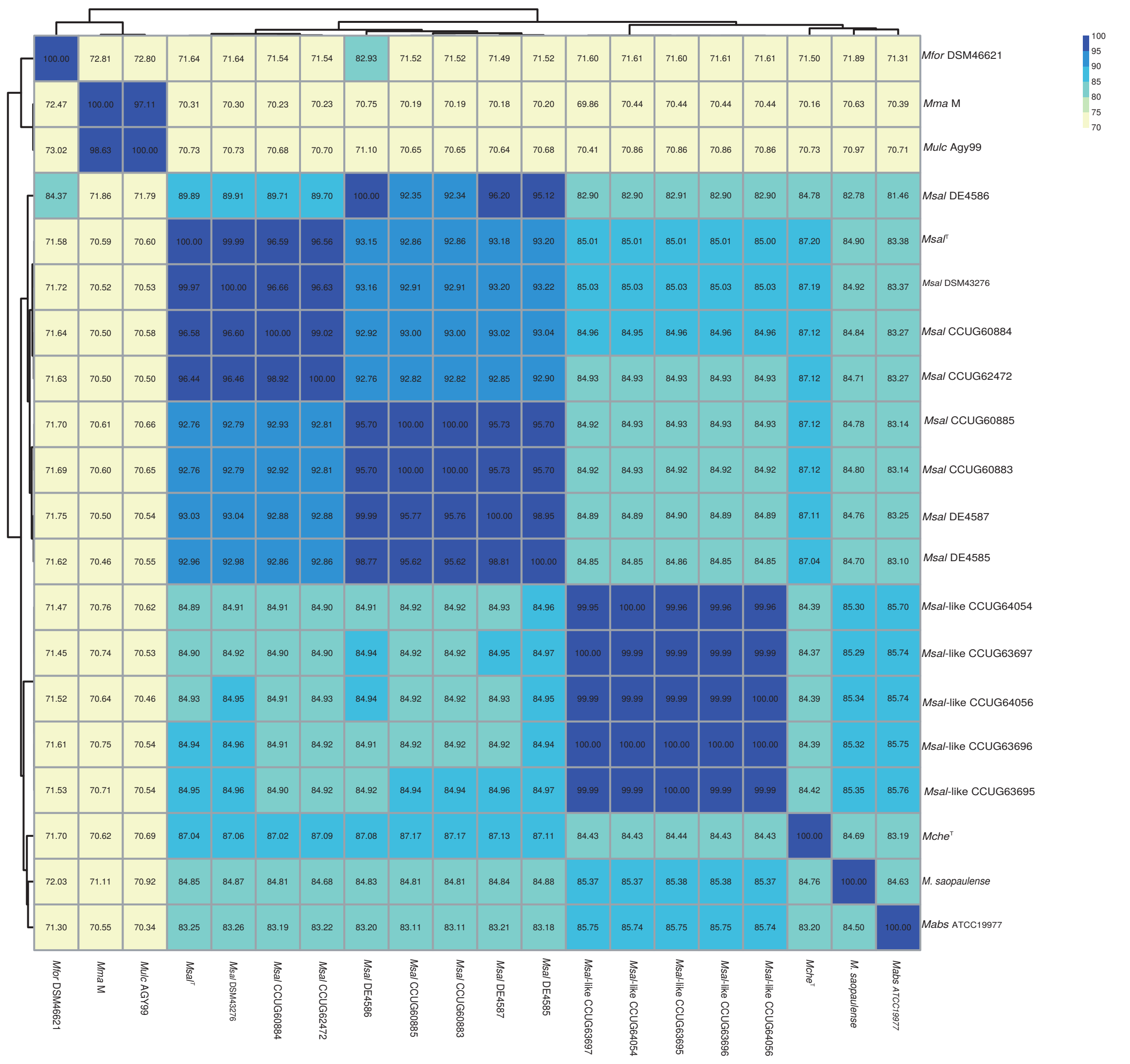

b.

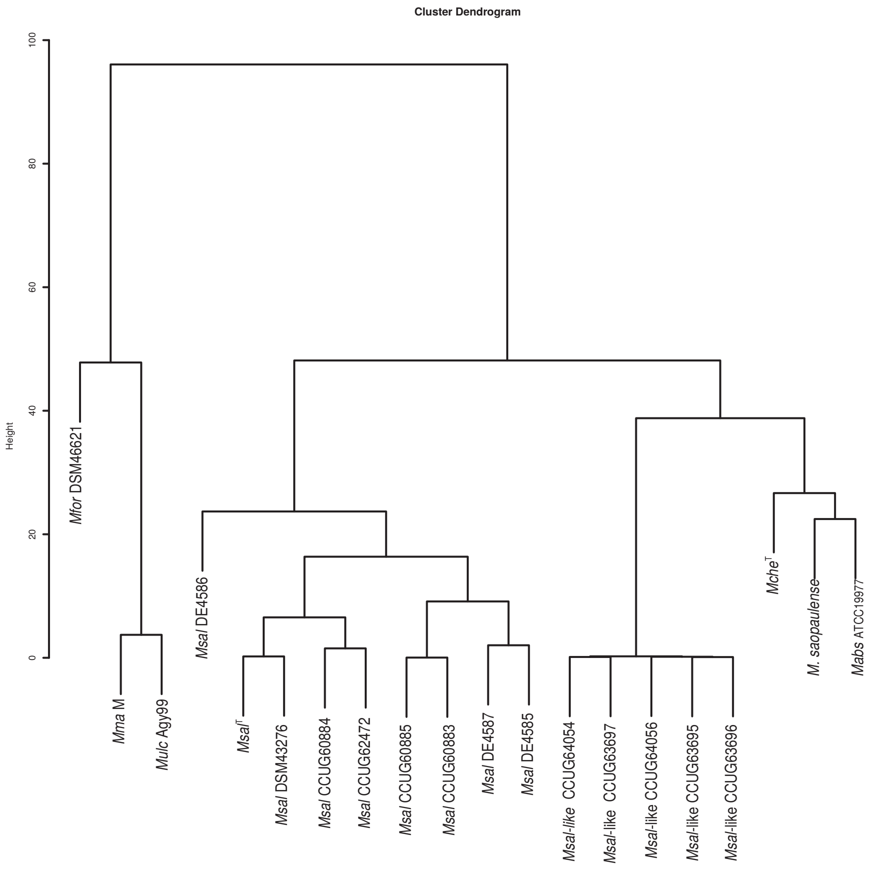

Supplement: Supplementary file 1 — Supplementary Information [file 41598_2019_40922_MOESM1_ESM.pdf]
